# RNA-binding protein CCDC137 activates AKT signaling and promotes hepatocellular carcinoma through a novel non-canonical role of DGCR8 in mRNA localization

**DOI:** 10.1186/s13046-023-02749-3

**Published:** 2023-08-05

**Authors:** Shuang Tao, Shu-Juan Xie, Li-Ting Diao, Guo Lv, Ya-Rui Hou, Yan-Xia Hu, Wan-Yi Xu, Bin Du, Zhen-Dong Xiao

**Affiliations:** 1 Biotherapy Center, The Third Affiliated Hospital, Sun Yat-Sen University, Guangzhou, 510630 P.R. China; 2 Present address: Guangdong Provincial Key Laboratory of Malignant Tumor Epigenetics and Gene Regulation, Guangdong-Hong Kong Joint Laboratory for RNA Medicine, Medical Research Center, Sun Yat-Sen Memorial Hospital, Sun Yat-Sen University, Guangzhou, 510120 P.R. China; 3 Institute of Vaccine, The Third Affiliated Hospital, Sun Yat-Sen University, Guangzhou, 510630 P.R. China; 4 Guangdong Key Laboratory of Liver Disease Research, The Third Affiliated Hospital, Sun Yat-Sen University, Guangzhou, 510630 P.R. China; 5 Department of Pathology, Shanghai First Maternity and Infant Hospital, School of Medicine, Tongji University, Shanghai, 200092 P.R. China

**Keywords:** RNA binding protein, Hepatocellular carcinoma, AKT, Cell proliferation, mRNA subcellular localization

## Abstract

**Background:**

RNA binding proteins (RBPs)—regulated gene expression play a vital role in various pathological processes, including the progression of cancer. However, the role of RBP in hepatocellular carcinoma (HCC) remains much unknown. In this study, we aimed to explore the contribution of RBP CCDC137 in HCC development.

**Methods:**

We analyzed the altered expression level and clinical significance of CCDC137 in database and HCC specimens. In vitro cell assays and in vivo spontaneous mouse models were used to assess the function of CCDC137. Finally, the molecular mechanisms of how CCDC137 regulates gene expression and promotes HCC was explored.

**Results:**

CCDC137 is aberrantly upregulated in HCC and correlates with poor clinical outcomes in HCC patients. CCDC137 markedly promoted HCC proliferation and progression in vitro and in vivo. Mechanistically, CCDC137 binds with *FOXM1*, *JTV1*, *LASP1* and *FLOT2* mRNAs, which was revealed by APOBEC1-mediated profiling, to increase their cytoplasmic localization and thus enhance their protein expressions. Upregulation of FOXM1, JTV1, LASP1 and FLOT2 subsequently synergistically activate AKT signaling and promote HCC. Interestingly, we found that CCDC137 binds with the microprocessor protein DGCR8 and DGCR8 has a novel non-canonical function in mRNA subcellular localization, which mediates the cytoplasmic distribution of mRNAs regulated by CCDC137.

**Conclusions:**

Our results identify a critical proliferation-related role of CCDC137 and reveal a novel CCDC137/DGCR8/mRNA localization/AKT axis in HCC progression, which provide a potential target for HCC therapy.

**Supplementary Information:**

The online version contains supplementary material available at 10.1186/s13046-023-02749-3.

## Introduction

RNA binding proteins (RBPs) are crucial regulators of gene expression. They interact with RNA molecules through specific binding domains [1] and play pivotal roles in almost all aspects of RNA metabolism such as transcription, splicing, localization, degradation and translation [2]. Due to the widespread regulatory networks of RBPs, their normal function is necessary for various biological processes, and dysfunction of RBPs can lead to several disorders and diseases, such as genetic diseases [2], cellular aging [3] and cancer [4, 5]. Accordingly, identifying the regulatory functionality of RBPs would promote the further understanding of human physiological and pathological activities.

Hepatocellular carcinoma (HCC) accounts for the majority of primary liver cancers and has a high potential for metastasis and poor prognosis [6]. In addition to traditional therapies, including surgery, chemotherapy and radiotherapy, molecular-targeted therapy has become a promising strategy [7]. However, due to the lack of efficient and reliable molecular targets, the application of molecular-targeted therapy still faces great challenges. Converging evidence shows that RBPs are associated with cancer development, including cancer proliferation [8], metastasis [9], dysregulated metabolism [10] and chemoresistance [11], and have become potential therapeutic targets for cancer therapy. RBPs have also been reported to contribute to hepatocarcinogenesis and serve as potential tools for diagnosis, prognosis and treatment [3, 12, 13]. Thus, a deeper understanding of HCC-related RBPs would shed new light on HCC treatment.

To explore the role of RBPs in HCC, we conducted large-scale data analysis using expression profiles from TCGA database and found that CCDC137 exhibited extensive differential expression. Previous studies have revealed that CCDC137 may play an important role in HCC progression [14, 15], but its specific function and mechanism are still unclear. In the present study, we found that CCDC137 is expressed at high levels in HCC tissues compared with that in adjacent normal hepatic tissue and could promote HCC in vitro and in vivo. Furthermore, we found that CCDC137 could activate the AKT signaling pathway through the upregulation of FOXM1, LASP1, JTV1, and FLOT2 protein levels. Mechanistically, CCDC137 could bind with *FOXM1*, *LASP1*, *JTV1*, and *FLOT2* mRNAs and increase their cytoplasmic localization through interaction with the microRNA biogenesis factor DGCR8. Overall, our results not only establish CCDC137 as a proliferation-related oncogenic RBP and a potential therapeutic target for HCC but also reveal a novel non-canonical function of DGCR8 in mRNA subcellular localization.

## Methods

### Expression and survival analysis

The expression analysis of CCDC137 in different cancer types and survival analysis of CCDC137 in HCC patients were performed using web-based tool GEPIA (http://gepia.cancer-pku.cn/) according to previously published article [16]. For the expression atlas of CCDC137 in different cancer types, Gene Expression Profile function was used. Related key parameters were set as follows: Differential Methods “ANOVA”; |Log2FC| Cutoff “0.5”; q-value Cutoff “0.01”; Log Scale “No”; Match TCGA normal and GTEx data. For CCDC137 expression in LIHC (liver hepatocellular carcinoma), Expression on Box Plots function was used. Related key parameters were set as follows: |Log2FC| Cutoff “0.5”; q-value Cutoff “0.01”; Log Scale “Yes”; Jitter Size “0.4”; Match TCGA normal and GTEx data. For survival analysis of CCDC137, Survival Plots function was used. Related key parameters were set as follows: Group Cutoff “Median”; Hazards Ratio (HR) “Yes”; 95% Confidence Interval “Yes”; Axis Units “Months”.

### HCC clinical samples

Human HCC tumor tissues and their paired non-cancerous hepatic tissues were recruited from 23 HCC patients of The Third Affiliated Hospital of Sun Yat-sen University (Guangzhou, China). The study was conducted with written informed consent of all patients and the approval of the Ethics Committee of The Third Affiliated Hospital of Sun Yat-sen University (Guangzhou, China).

### Cell culture

HEK293T cells, Human HCC cell line Huh7, HCCLM3, JHH-7 and PLC/PRF/5 were purchased from the Chinese Academy of Sciences Cell Bank (Shanghai, China). HEK293T, Huh7, HCCLM3, JHH-7 were cultured with Dulbecco's modified Eagle's medium (DMEM) (Gibco) containing 10% (vol/vol) Fetal Bovine Serum (FBS) (ExCell Bio, FSP500) and 1% (vol/vol) Penicillin–Streptomycin (P/S) (KeyGen, KGY0023). PLC/PRF/5 were cultured with Minimum Essential Medium (MEM, Gibco) containing 10% (vol/vol) FBS, 1% (vol/vol) Non-Essential Amino Acids Solution (NEAA) (Gibco), 1% (vol/vol) L-Glutamine (Gibco) and 1% (vol/vol) P/S. All the cells were incubated in a humidified-atmosphere incubator of 5% CO_2_ at 37 °C.

### Plasmids and stable cell lines

PCR-amplified human CCDC137 was cloned into lentiviral plasmid pCDH-V5-P2A-puromycin to generate CCDC137 overexpression lentiviral plasmid. PCR-amplified human DGCR8^773^ (full length) and truncated DGCR8^700^ were cloned into lentiviral plasmid pCDH-blasticidin to generate DGCR8^773^ and DGCR8^700^ overexpression lentiviral plasmid. sgRNA target at human CCDC137 was cloned into lentiviral plasmid pLV-sgRNA-dCas9-KRAB-T2A-PuroR to generate human CCDC137 knock-down lentiviral plasmid. sgRNAs target at FOXM1, JTV1, LASP1 and FLOT2 were cloned into lentiviral plasmid pLenti6.3-spCas9-sgRNA-blasticidin to generate FOXM1, JTV1, LASP1 and FLOT2 knock-down plasmids, respectively. Plasmids were extracted using Plasmid Mini Kit II (Omega, D6945-02). Lentiviruses were produced by co-transfecting HEK293T cells with lentiviral plasmids and the equal amounts of packaging plasmids (psPAX2: pMD2.G = 3:1) using polyethyleneimine (PEI, Polysciences). After 48 h, lentivirus supernatant was harvested by centrifuging at 3,500 rpm for 5 min and filtered through a 0.45-μm pore size filter. Cells were infected with lentivirus and selected with puromycin (Invivogen) or blasticidin (Invivogen). The sequences of CDS and gRNAs used are listed in Supplementary Table.

### Small interfering RNA (siRNA) transfection

Two small interfering RNA of DGCR8 (stB0001678B, stB0001678C) and negative control (siN0000001-1–5) were purchased from Guangzhou RiboBio Co., Ltd. Lipofectamine 2000 (Invitrogen) was used to transfect siRNAs at 100 nM concentration into target cells following manufacturer's instruction. After 48 h transfection, cells were harvested and subjected to subsequent analysis.

### Colony formation assay

Cells were seeded in 12-well plates at a density of 10,000 cells/well and incubated in 5% CO_2_ at 37℃ for 0, 2, 4 and 6 days, respectively. Cells were washed with PBS once, fixed with methanol for 10 min and then stained with 0.1% (vol/vol) crystal violet in PBS for 10 min, then photographed using a digital camera. The colony formation was measure by the absorbance at OD570 after dissolved by 33% (vol/vol in PBS) acetic acid.

### Spheroid formation assay

Cells were cultured at a density of 10,000 cells/well in serum-free DMEM/ F12 (1:1) medium (HyClone, SH30272.02), supplemented with 1% P/S, 20 ng/mL recombinant human epidermal growth factor (EGF) (Sigma), 10 ng/mL recombinant human basic fibroblast growth factor (bFGF) (R&D Systems) and B27 (1:50, Gibco). Cells were cultured in six-well ultra-low adherent plates and maintained in 5% CO_2_ at 37℃ for 5–10 days. Cells were replenished with 500μL supplemented medium every second day.

### RNA isolation and real-time quantitative RT‒PCR (RT-qPCR)

Total RNA was extracted from cells with TRIzol reagent (Life, 265,709, CA, USA). First-strand cDNA for PCR analyses was synthesized with HiScript III RT SuperMix for qPCR (+ gDNA wiper) (Vazyme, R323-01). RT-qPCR was performed using ChamQ Universal SYBR qPCR Master Mix (Vazyme, Q711-02) and was run on Roche LightCycler 480. Human GAPDH gene served as an internal control. The RT-qPCR results were analyzed as relative RNA levels of the cycle threshold (CT) values, which were then converted into fold change. Results are presented as the means ± SD. All primers for RT-qPCR are listed in Supplementary Table.

### Western blot

For cultured cells, cells were harvested and lysed in ice-cold Enhanced RIPA Lysis Buffer (Shanghai Wansheng Haotian Biological Technology, EZPS03-1) containing Phosphatase Inhibitor and Protease Inhibitor Cocktail Tablets (Roche). For mice liver tissues, tissues were lysed in RIPA lysis buffer adding protease inhibitor (Roche) and phosphatase inhibitor (Roche) and grinded using a Homogenizer (Servicebio). Protein concentration was determined by BCA protein quantification assay kit (KeyGEN). Equivalent 30 μg total protein extracts were separated by SDS–PAGE and then transferred to nitrocellulose membranes (Merck Millipore). The membranes were blocked with 5% non-fat dry milk in TBST for 1 h at room temperature. Then incubated in the corresponding primary antibodies overnight at 4℃. The next day, after three times washing, membranes were incubated with secondary antibodies (diluted at 1:5000 with primary antibody dilution) at room temperature for 1 h. Immunoreactivities were determined using WesternBright ECL Western blotting detection kit (Advansta). The following antibodies were used in this study: Vinculin (Proteintech, 26520-1-AP, 1:1000 dilution), CCDC137 (Proteintech, 27201–1-AP, 1:1000 dilution), JAK1 (Cell Signaling Technology, #3332, 1:1000 dilution), phospho-JAK1 (Cell Signaling Technology, #3331, 1:1000 dilution), STAT1 (Abcam, ab31369, 1:1000 dilution), phospho Y701-STAT1 (Abcam, ab30645, 1:1000 dilution), STAT3 (Cell Signaling Technology, #12640, 1:1000 dilution), phospho Tyr705-STAT3 (Cell Signaling Technology, #9145, 1:1000 dilution), GSK3β (Cell Signaling Technology, #5676, 1:1000 dilution), phospho-GSK3β (Cell Signaling Technology, #5558, 1:1000 dilution), ERK (Cell Signaling Technology, #4695, 1:4000 dilution), phospho-ERK (Cell Signaling Technology, #4370, 1:4000 dilution), S6K (Cell Signaling Technology, #9262, 1:1000 dilution), phospho-S6K (Santa Cruz, sc-8418, 1:1000 dilution), S6 (Cell Signaling Technology, #2217, 1:2000 dilution), phospho-S6 (Cell Signaling Technology, #5364, 1:2000 dilution), JNK (Cell Signaling Technology, #9252, 1:2000 dilution), phospho-JNK (Cell Signaling Technology, #9251, 1:2000 dilution), YAP (Cell Signaling Technology, #14074, 1:1000 dilution), phospho-YAP (Cell Signaling Technology, #4911, 1:1000 dilution), AKT (Cell Signaling Technology, #4691, 1:1000 dilution), Ser473 phospho-AKT (Santa Cruz, sc-7985, 1:1000 dilution), Thr308 phospho-AKT (Cell Signaling Technology, #4056, 1:1000 dilution), Lamin A/C (Cell Signaling Technology, #2032, 1:1000 dilution), GFP (ABclonal, AE011, 1:4000 dilution), FOXM1 (Proteintech, 13147-1-AP, 1:1000 dilution), JTV1 (Proteintech, 10424-1-AP, 1:1000 dilution), LASP1 (Proteintech, 10515-1-AP, 1:1000 dilution), FLOT2 (Proteintech, 28208-1-AP, 1:1000 dilution), DGCR8 (Proteintech, 10996-1-AP, 1:1000 dilution), β-actin (Bioworld, AP0060, 1:1000 dilution), Flag (Sigma, F1804, 1:1000 dilution), V5 (Sigma, V8012, 1:1000 dilution). Secondary antibodies for Rabbit (#7074 s, 1:5000) and Mouse (#7076 s, 1:5000) were obtained from Cell Signaling Technology. The quantification of protein levels in Western Blot analysis were performed using Image J and the bar graphs were generated by GraphPad Prism 8.3.0.

### Transcriptome-sequencing and small RNA-sequencing

The transcriptome-sequencing, small RNA-sequencing and data analysis were provided by LC-Bio (Hangzhou, China). Total RNA was extracted with TRIzol reagent (Life, 265709, CA, USA). Bioanalyzer 2100 and RNA 6000 Nano LabChip Kit (Agilent, CA, USA, 5067–1511) was used to determine the quantity and purity of total RNA and RNA samples with RIN number > 7.0 were choosed to construct sequencing library. For mRNA-sequencing, Dynabeads Oligo (dT) (ThermoFisher, CA, USA) was used to purify mRNA and then mRNA was fragmented using divalent cations under (Magnesium RNA Fragmentation Module, NEB, e6150, USA) under 94℃ 5-7 min. Then RNA fragments were transcribed into cDNA using SuperScript™ II Reverse Transcriptase (Invitrogen, 1896649, USA) and converted into U-labeled second-stranded DNAs using E. coli DNA polymerase I (NEB, m0209, USA), RNase H (NEB, m0297, USA) and dUTP Solution (Thermo Fisher, R0133, USA). 2 × 150 bp paired-end sequencing (PE150) of the cDNA library was performed by Illumina Novaseq™ 6000 following the vendor's recommended protocol. For small RNA-sequencing, TruSeq Small RNA Sample Prep Kits (Illumina, San Diego, USA) was used to prepare small RNA library and then single-end sequencing (1 × 50 bp) was performed by an Illumina Hiseq2500 following the vendor’s recommended protocol. The genes with false discovery rate (FDR) below 0.05 and absolute fold change ≥ 2 were considered differentially expressed genes. Advanced heat plots and volcano plots were performed using the OmicStudio tools at https://www.omicstudio.cn/tool. Venn plots were performed at https://bioinformatics.psb.ugent.be/webtools/Venn/. The raw and processed data of transcriptome-sequencing and small RNA-sequencing in CCDC137-overexpressing cells and CCDC137-knockdown cells can be found Gene Expression Omnibus (GEO) under accession code GSE218087.

### STAMP-sequencing

STAMP was carried out as previously described [17]. The high-throughput sequencing was performed by Guangzhou Huayin Health Medical Group CO.,Ltd. (Guangzhou, China). The fusion protein of GFP-APOBEC1 and CCDC137-APOBEC1 were constructed into pCDH-TRE3G-Tet-On 3G plasmid. Cells were transfected with GFP-APOBEC1 and CCDC137-APOBEC1 with or without doxycycline treatment were harvested. Total RNA was extracted with TRIzol reagent (Life, 265709, CA, USA). After the quality inspection using Agilent 2100 Bioanalyzer (Agilent, G2939AA, CA, USA) and NanoPhotometer® (Implen, N60, Munich, Germany), mRNA with poly(A) is purified with VAHTS® mRNA Capture Beads with Oligo (dT) (Vazyme, N401-01, Nanjing, China). VAHTS® Universal V6 RNA-seq Library Prep Kit (Vazyme, NR604, Nanjing, China) was used to fragment RNA under 94℃ 8 min and then mRNA fragments were reversed transcribed into cDNA. After UDG enzyme treatment, size selection by VAHTS® DNA Clean Beads (Vazyme, N411, Nanjing, China) amplification and purification, 2 × 150 bp paired-end sequencing (PE150) was performed by Illumina Novaseq™ 6000 system (Illumina Corporation, San Diego,USA) following the vendor's recommended protocol. C to U mutation sites analysis was conducted as previously published [17, 18]. Reads duplicates were collapsed using fastq2collapse.pl from CTK and aligned to Ensembl-hg19 using BWA.aln. C to U mutations were acquired by joinWrapper.py and calculated by CIMS.pl. Then, C to U sites were further filtered and only the sites that had FDR <  = 1, mutation frequency >  = 2, tag counts >  = 10, and mutation frequency/ tag counts (mutation ratio) ranges from 0.1 to 0.6 were kept. Genes with mutation sites only found in CCDC137-APOBEC1 expressing cells were subjected to further analysis. The raw and processed data of STAMP-sequencing can be found in Gene Expression Omnibus (GEO) under accession code GSE218087.

### RNA immunoprecipitation (RIP)

Cells were harvested by trypsin and crosslinked with 0.75% formaldehyde followed by 1.25 M glycine quenching. Then cells were lysed in ice-cold Enhanced RIPA Lysis Buffer containing Phosphatase Inhibitor, Protease Inhibitor, Recombinant RNase Inhibitor (Accurate Biology, AG11608) and sonicated for 20 min with a 5 s on / 5 s off cycle at 80% power by sonicator (SCIENTZ 08-III). The lysates were immunoprecipitated with Anti-V5 Affinity Gel (GNI4510-V5) or S-protein Agarose (Novagen, 69704) overnight at 4℃. Cell lysates and immunoprecipitants were digested with DNase I (RNase Free) (Accurate Biology, AG12001), RNase Inhibitor at 37℃ 1,200 rpm rotation for 30 min; and digested with Proteinase K (Accurate Biology, AG12004) at 60℃ 1,200 rpm rotation for 30 min. RNA was purified using MicroElute RNA Clean-Up Kit (Omega) and then detected by RT-qPCR.

### Co-immunoprecipitation (CO-IP)

PCR-amplified GFP was cloned into plasmid pCDH-V5-P2A-puromycin to generate V5-tagged GFP plasmid. PCR-amplified DGCR8 and GFP were cloned into plasmid pCDH-SFB to generate SFB-tagged DGCR8 and GFP plasmids. Cells transfected with corresponding plasmids were harvested by trypsin digestion and washed twice with PBS. Cell pellets were lysed in ice-cold Enhanced RIPA Lysis Buffer containing Phosphatase Inhibitor, Protease Inhibitor and Benzonase Nuclease (HaiGene, C2001). Protein concentration was determined by BCA protein quantification assay kit (KeyGEN). Equivalent protein was immunoprecipitated using Normal Rabbit IgG (Cell Signaling Technology, #2729, 2 μg), CCDC137 (Proteintech, 27201–1-AP, 2 μg), Anti-V5 Affinity Gel (GNI4510-V5) or S-protein Agarose (Novagen, 69704). Cell lysates and immunoprecipitants were detected by Western blot using corresponding antibodies.

### Subcellular fractionation

Cells were harvested by trypsin digestion and washed twice with PBS. Cell pellets were resuspended in 200μL ice-cold cytoplasmic lysis buffer (0.15% NP-40, 10 mM Tris PH 7.4, 150 mM NaCl in DEPC water) and incubated on ice for 5 min. Then the lysate was transferred onto 500μL ice-cold sucrose buffer (10 mM Tris PH 7.4, 150 mM NaCl, 24% sucrose in DEPC water), and spun at 1,3000 rpm, 4℃ for 10 min. The supernatant was collected as cytoplasmic fraction. 1/10 (70μL) was saved for RNA isolation. The nuclear pellet was resuspended in 200μL ice-cold cytoplasmic wash buffer (10 mM Tris PH 7.4, 150 mM NaCl in DEPC water) and passed through 500μL ice-cold sucrose buffer again. The washed nuclear pellet was then resuspended in 200μL ice-cold nuclei lysis buffer (20 mM HEPES PH 7.4, 7.5 mM MgCl_2_, 0.2 mM EDTA, 0.3 M NaCl, 1 M urea, 1% NP-40, 1 mM DTT in DEPC water) and the vortex vigorously for 5 s, incubated on ice for 1 min and then spun at 1,4000 rpm, 4℃ for 2 min. The supernatant was collected as nuclear fractionation and 1/5 (40μL) was saved for RNA isolation. Subsequent digestion, RNA purification and detection has already described in RIP.

### Murine HCC model

Male C57BL/6J mice were purchased from Gem Pharmatech (Nanjing, China) at 4 weeks of age and allowed to acclimate for 1 week prior to the start of the study. 5 mice were randomly grouped. Plasmid pT3-EF1α-c-Myc was a gift from Dr. Xin Chen lab. Human CCDC137 was amplified and cloned into plasmid pT3-EF1α-cMyc to generate pT3-EF1α-cMyc-T2A-hCCDC137 plasmid. sgRNA targeted at P53, nontarget and mouse CCDC137 was cloned into pX330-Cas9 and pX333-Cas9 to generate pX330-Cas9-sgP53, pX333-Cas9-sgP53-sgnontarget and pX333-Cas9-sgP53-sgccdc137 plasmids. 20 μg pT3-EF1α-cMyc, 20 μg pX330-Cas9-sgP53, 1.6 μg pCMV-SB transposase were mixed as control group of CCDC137-overexpression; 20 μg pT3-EF1α-cMyc-P2A-hCCDC137, 20 μg pX330-Cas9-sgP53, 1.6 μg pCMV-SB transposase were mixed as CCDC137-overexpression group; 20 μg pT3-EF1α-cMyc, 20 μg pX333-Cas9-sgP53-sgnontarget, 1.6 μg pCMV-SB transposase were mixed as control group of ccdc137-knockout group and 20 μg pT3-EF1α-cMyc, 20 μg pX333-Cas9-sgP53-sgccdc137, 1.6 μg pCMV-SB transposase were mixed as ccdc137-knockout group. The mixture of plasmids for each group were mixed in 2 mL saline and then hydrodynamic injected into tail vein of mice alternately between two groups in each experiment within 15 s. Mice were sacrificed at 4–5 weeks after hydrodynamic injection when they start to exhibit distended abdomen or decreased locomotor activity. Detailed information about hydrodynamic tail vein injection assay can be found at https://pharm.ucsf.edu/xinchen/protocols/hydrodynamic-tail-injection. All mice were reared and all experiments were performed at the animal facility of Jennio Biotech Co., Ltd (Guangzhou, China). Animal ethical approval were granted by the Institutional Animal Care and Use Committee (IACUC), Jennio Biotech Co., Ltd.

### Immunohistochemistry

The tissues were fixed in 4% paraformaldehyde, embedded with paraffin, and sectioned into 5 μm-thick slices. Following deparaffinized in xylene, the slices were dehydrated in a 100, 95, 85, 75% ethanol gradually, antigen retrieved under high pressure with citrate buffer, immersed in 3% hydrogen peroxide solution and blocked by Blocking Buffer for Immunol Staining (Beyotime). Slices were incubated with the corresponding primary antibodies at 4 °C overnight and dyed with Peroxidase/DAB (Dako, K5007). Finally, the slices were re-dyed with hematoxylin for imaging. The following antibodies were used: CCDC137 (Proteintech, 27201-1-AP, 1: 100 dilution), Ki-67 (Abcam, ab15580, 1: 100 dilution), phospho Ser473-AKT (Proteintech, 66444-1-Ig, 1: 100 dilution), phospho Thr308-AKT (Proteintech, 29163-1-AP, 1: 100 dilution). The area percentage of positive staining was calculated by Image J software and subjected to statistical analysis.

### Immunofluorescence staining

The growing cells were plated into Miliicell EZ slide (Millipore, PEZGS0816) overnight. Cells were fixed with 4% paraformaldehyde for 15 min, permeabilized with 0.5% TritonX-100 (Sigma, T8787) in PBS for 5 min, blocked with 3% BSA at room temperature for 1 h and incubated with primary antibody at 4 °C overnight. The following antibodies were used: CCDC137 (Proteintech, 27201-1-AP, 1: 100 dilution), DGCR8 (Proteintech, 10996-1-AP, 1: 100 dilution), V5 (Sigma, V8012, 1:200 dilution). After rinsing with PBS, the cells were incubated with Alexa Fluor Highly Cross-Adsorbed Secondary Antibody (Invitrogen, A11032, A11034) for 1 h at room temperature, followed by staining with DAPI. Images were acquired using Zeiss LSM880 laser scanning confocal microscope.

### Statistical analysis

The values were presented as mean ± standard deviation (s.d.) of at least three independent experiments. Statistical analysis was performed using two-tailed Student’s *t*-test by GraphPad Prism 8.3.0. No statistical method was used to predetermine sample size. None of the samples/animals was excluded from the experiment. For all statistical analysis, differences were considered as statistically significant at values of *p < 0.05, ** p < 0.01, *** p < 0.001, **** p < 0.0001.

## Results

### CCDC137 expression is elevated and associated with patient prognosis in HCC

**Fig. 1:**
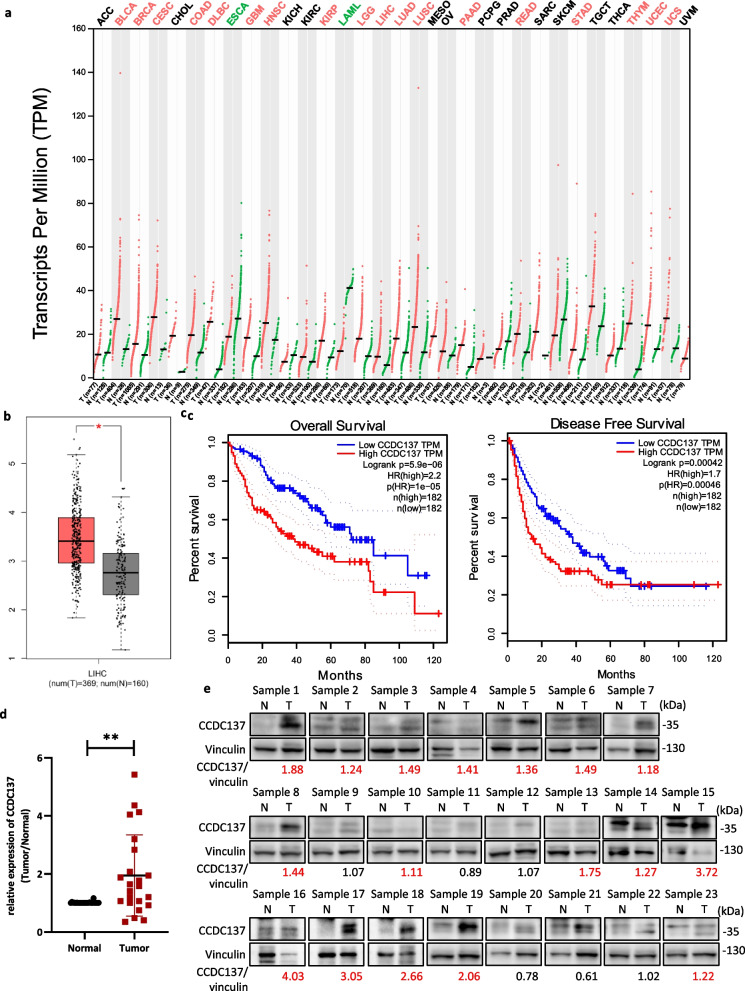
CCDC137 expression is elevated and associated with patient prognosis in HCC. **a**, **b**, **c** Analysis of CCDC137 expression from GEPIA (http://gepia.cancer-pku.cn/). **a** Dot plot shows CCDC137 expression in tumor samples (T, red) and paired normal tissues (N, green) across various tumor types. The X axis is the number of T and N for each tumor type. The Y axis is transcripts per million (TPM). Each dot represents expression level of samples. Labels in the top of the figure show different types of tumors marked with different color. Red, CCDC137 expression in T is significantly higher than N; green, CCDC137 expression in T significantly lower than N; black, have no significant difference. (log_2_FC cutoff = 0.5, *p* value cutoff = 0.01) (**b**) Box plot analysis of CCDC137 expression in 369 HCC tumor samples (T) and 160 normal tissues (N) (p* < 0.05). (log_2_FC cutoff = 0.5, *p* value cutoff = 0.01) (**c**) Kaplan–Meier survival analysis of the correlation between CCDC137 expression and overall survival and disease-free survival in HCC. **d** *CCDC137* mRNA levels in 23 pairs of HCC tumor samples and corresponding adjacent non-tumor tissues. Data were presented as mean ± s.d. of *n* = 3 independent experiments. *p* value: ^∗∗^*p* < 0.01 by paired Student's *t*-test. **e** CCDC137 protein levels in 23 pairs of HCC tumor samples and corresponding adjacent non-tumor tissues. The fold change of CCDC137/Vinculin ratio in tumor samples to normal samples over 1.1 were marked in red

To explore the expression profile of the uncharacterized RBP CCDC137 in cancers, a web-based tool GEPIA (Gene Expression Profiling Interactive Analysis) based on RNA-seq data from The Cancer Genome Atlas (TCGA) and the GTEx projects [16] were used. CCDC137 exhibits abnormal expression in cancers, with elevated expression in tumor tissues compared with that in normal tissues in most cancer types, including HCC (Fig. 1a, b). Kaplan–Meier survival analysis showed that patients with high CCDC137 expression had shorter overall and disease-free survival than patients with low CCDC137 expression (Fig. 1c).

To confirm its dysregulation in HCC, we then investigated CCDC137 expression in clinical specimens. Primary tumor tissues and paired noncancerous hepatic tissues were collected from twenty-three HCC patients. By detecting the mRNA and protein expression levels of CCDC137 in these specimens, we found that CCDC137 expression was increased in most (17/23) HCC tumor tissues compared with that in the surrounding normal tissues (Fig. 1d, e), which verified the expression from the databases. Collectively, these data demonstrate that CCDC137 expression is significantly elevated in HCC tumor tissues and suggest that CCDC137 may be an oncogenic factor and a potential predictive factor of poor prognosis in HCC.

### CCDC137 promotes HCC anchorage-dependent and anchorage-independent proliferation in vitro

**Fig. 2:**
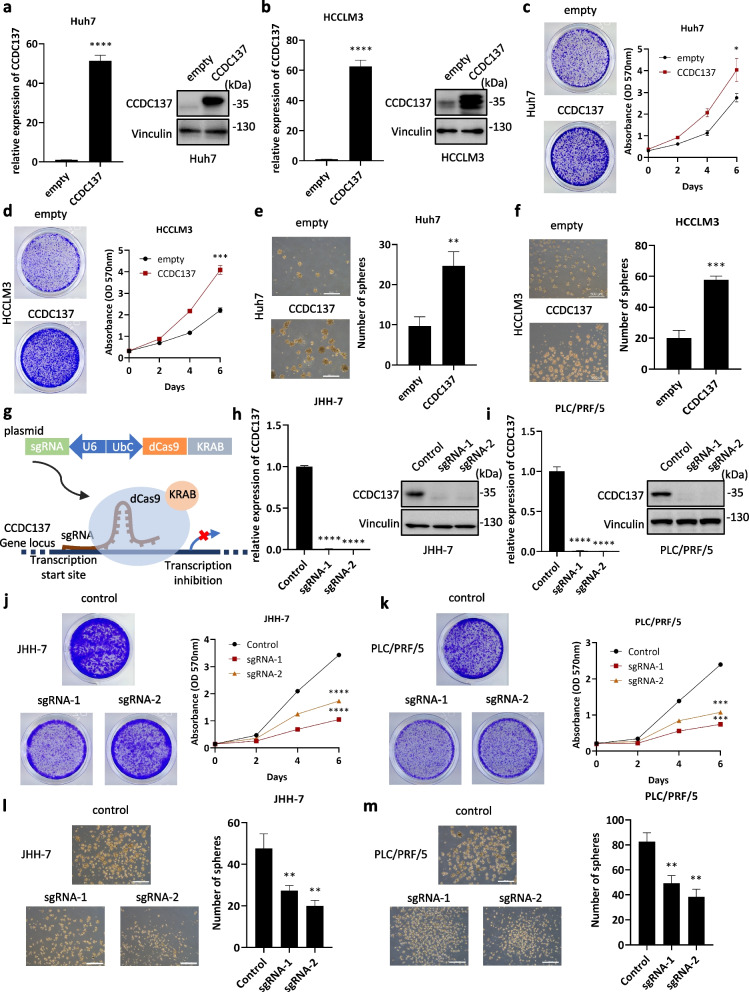
CCDC137 promotes HCC anchorage-dependent and anchorage-independent proliferation in vitro. **a**, **b** The mRNA and protein levels of CCDC137 in Huh7 (**a**) or HCCLM3 (**b**) cells stably overexpressing CCDC137. **c**, **d** Cell anchorage-dependent proliferation was measured by crystal violet staining and quantified with the absorbance at OD 570 nm in Huh7 (**c**) and HCCLM3 (**d**) cells stably overexpressing CCDC137. **e**, **f** Cell anchorage-independent proliferation was measured by spheroid formation assay in Huh7 **e** and HCCLM3 (**f**) cells stably overexpressing CCDC137. **g** The schematic illustration of CRISPRi. **h**, **i** The mRNA and protein levels of CCDC137 in CCDC137 stably knocked-down JHH-7(h) or PLC/PRF/5 (**i**) cell lines. **j**, **k** Cell anchorage-dependent proliferation was measured by crystal violet staining and quantified with the absorbance at OD 570 nm in CCDC137 stably knocked-down JHH-7(j) and PLC/PRF/5(k) cell lines. **l**, **m** Cell anchorage-independent proliferation was measured by spheroid formation assay in CCDC137 stably knocked-down JHH-7(l) and PLC/PRF/5(m) cell lines. Scale bars: 500 μm (**e**, **f**, **l**, **m**). Data were presented as mean ± s.d. of *n* = 3 independent experiments. *p* value: ^∗^*p* < 0.05, ^∗∗^*p* < 0.01, ^∗∗∗^*p* < 0.001, ^∗∗∗∗^*p* < 0.0001 by Student's *t*-test

We first examined the expression level of CCDC137 in normal hepatocyte cell lines and HCC cell lines (Supplementary Figure S1). To examine the roles of CCDC137 in hepatocarcinogenesis, we stably overexpressed CCDC137 in Huh7 and HCCLM3 cells using a lentiviral vector with the empty vector as a negative control (Fig. 2a, b). Using crystal violet staining and spheroid formation assays to evaluate of cell anchorage-dependent and anchorage-independent proliferation properties, respectively, we found that ectopic expression of CCDC137 significantly promoted HCC cell proliferation (Fig. 2c-f).

To further validate the role of CCDC137 in HCC cells, we stably knocked down CCDC137 expression using two guide RNAs (gRNAs) via CRISPR inhibition (CRSPRi) (Fig. 2g). Both gRNAs were targeted downstream of the transcription start sites of CCDC137 and guided the inactive Cas9 (dCas9) combined with the Krüppel-associated box (KRAB) repressor to inhibit the transcription of CCDC137 (Fig. 2h, i). As expected, depletion of CCDC137 significantly inhibited PLC/PRF/5 and JHH-7 cell anchorage-dependent and anchorage-independent proliferation, as indicated by crystal violet staining and spheroid formation assays, respectively (Fig. 2j-m). Collectively, these results showed that CCDC137 is an oncogenic protein in hepatocarcinogenesis that can promote anchorage-dependent and anchorage-independent proliferation of HCC cells in vitro.

### CCDC137 promotes HCC development in vivo

**Fig. 3:**
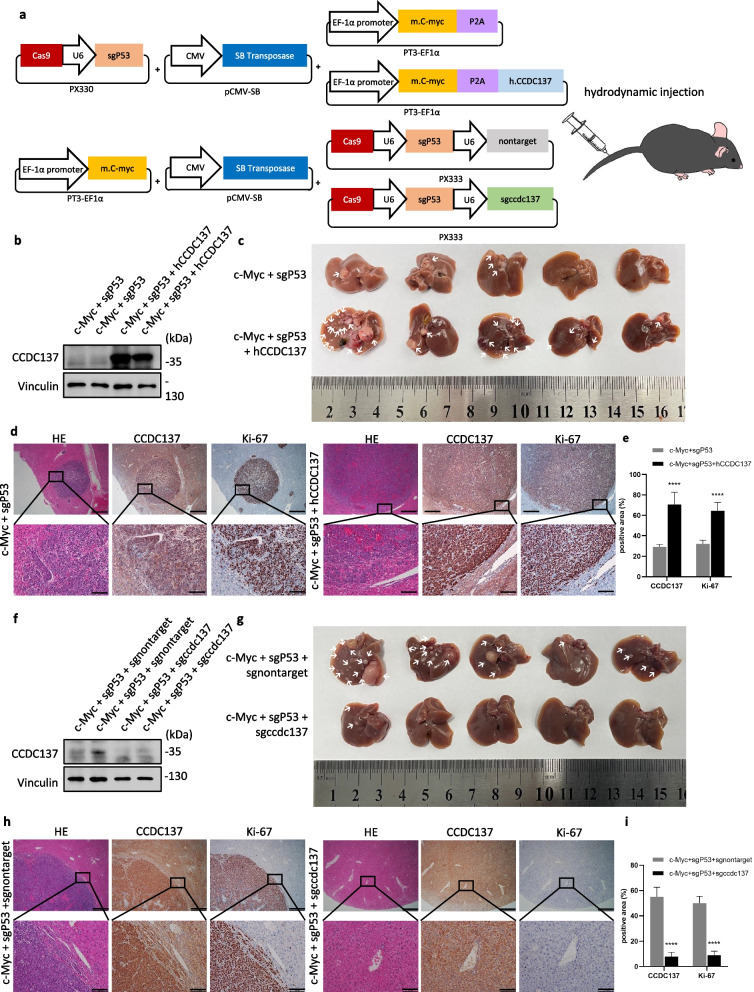
CCDC137 promotes HCC proliferation in vivo. **a** Schematic illustration of hydrodynamic injection to generate murine hepatocyte-derived HCC model. Plasmid expressing sgP53, SB transposase, c-myc with or without human CCDC137 were injected to mice to examine the effect of CCDC137 overexpression. Plasmid expressing c-myc, SB transposase, sgP53 with or without sgRNA targeted mouse CCDC137 were injected to mice to examine the effect of CCDC137 depletion. **b**-**d** Effects of CCDC137 overexpression in HCC mouse model. **b** The protein levels of CCDC137 in CCDC137-overexpressing mice and control mice (*n* = 2). The mice were sacrificed at 4–5 weeks after injection and the livers were excised (**c**). *n* = 5 mice in each group. **d** H&E staining and IHC staining for CCDC137 and Ki-67 in CCDC137-overexpressed mice and control mice. Scale bars: 500 μm (upper panels), 100 μm (lower panels). **e** Comparison of CCDC137 and Ki-67 positive area. **f-i** Effects of CCDC137 knockdown in HCC mouse model. **f** The protein levels of CCDC137 in CCDC137-knockdown mice and control mice (*n* = 2). The mice were sacrificed at 4–5 weeks after injection and the livers were excised (**g**). *n* = 5 mice in each group. **h** H&E staining and IHC staining for CCDC137 and Ki-67 in CCDC137-knocked down mice and control mice. **i** Comparison of CCDC137 and Ki-67 positive area. Scale bars (**d**, **g**): 500 μm (upper panels), 100 μm (lower panels). Data were presented as mean ± s.d. *p* value: ^∗∗∗∗^*p* < 0.0001 by paired Student's *t*-test

We next confirmed the role of CCDC137 in hepatocarcinogenesis in vivo. We employed a murine hepatocyte-derived HCC model by overexpressing the c-Myc proto-oncogene in the context of deficiency of the tumor suppressor p53 [19, 20]. Plasmids expressing c-Myc and human CCDC137 or carrying single guide RNAs targeting p53 and murine CCDC137 were constructed and then hydrodynamically injected through the tail vein of C57BL/6 mice (Fig. 3a). The results showed that overexpression of CCDC137 significantly promoted HCC tumorigenesis, as CCDC137-overexpressing mice developed more and larger tumors on the liver surface (Fig. 3b, c). Hematoxylin and eosin (H&E) staining revealed larger areas of tumors in the livers of CCDC137-overexpressing mice than in those of control mice (Fig. 3d). Additionally, CCDC137 and Ki-67 immunohistochemistry (IHC) staining further verified that the overexpression of CCDC137 promoted HCC proliferation (Fig. 3d, e). Conversely, CCDC137-knockout mice developed fewer and smaller tumors on the liver surface than those in control mice (Fig. 3f, g). H&E staining and IHC staining confirmed that the depletion of CCDC137 could suppress HCC tumorigenesis and proliferation (Fig. 3h, i). Collectively, these data demonstrate that CCDC137 promotes HCC tumorigenesis in vivo and may serve as a potential therapeutic target.

### CCDC137 promotes HCC cell proliferation through activation of AKT signaling

**Fig. 4:**
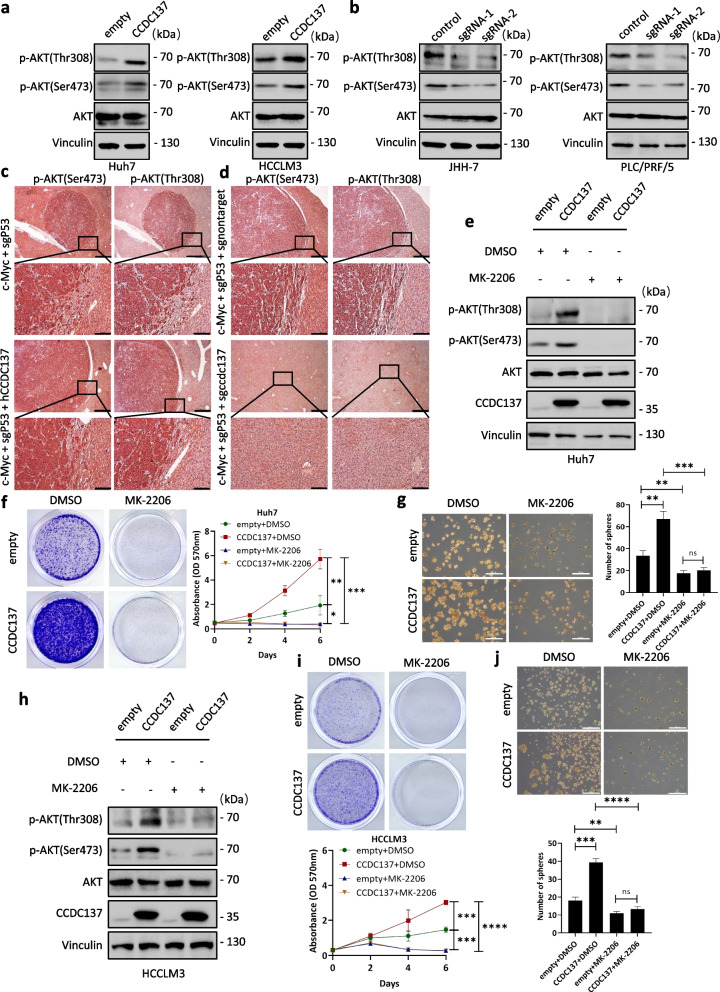
CCDC137 promotes HCC proliferation through activation of AKT signaling. **a** AKT phosphorylation level at Ser473 and Thr308 in CCDC137-overexpressing Huh7 and HCCLM3 cells. **b** AKT phosphorylation level at Ser473 and Thr308 in CCDC137-knockdown PLC/PRF/5 and JHH-7 cells. IHC staining for AKT phosphorylation at Ser473 and Thr308 in CCDC137-overexpressing (**c**) and CCDC137-knockout (**d**) mice. Scale bars (**c**, **d**): 500 μm (upper panels), 100 μm (lower panels). AKT phosphorylation level at Ser473 and Thr308 after AKT inhibitor MK-2206 treatment (5 μM, 48 h) in CCDC137-overexpressing Huh7 (**e**) and HCCLM3 cells (**h**). Cell proliferation was detected by crystal violet staining (**f**, **i**) and spheroid formation assay (**g**, **j**) after AKT inhibitor MK-2206 treatment (5 μM, 48 h) in CCDC137-overexpressing Huh7 and HCCLM3 cells. Scale bars (**g**, **j**): 500 μm. Data were presented as mean ± s.d. of *n* = 3 independent experiments. *p* value: ^∗^*p* < 0.05, ^∗∗^*p* < 0.01, ^∗∗∗^*p* < 0.001, ^∗∗∗∗^*p* < 0.0001 by paired Student's *t*-test

To gain insights into the molecular mechanism underlying the oncogenic role of CCDC137, we examined several downstream signaling pathways in CCDC137-overexpressing and CCDC137-knockdown cell lines. The signaling pathways detected are known to regulate HCC proliferation such as JAK/STAT, GSK-3β, ERK, S6K/S6, JNK, YAP and AKT [21–24] (Supplementary Figure S2). Among the signaling pathways examined, we found that only the phosphorylation level at AKT phosphorylation sites Ser473 and Thr308 were significantly increased in CCDC137-overexpressing Huh7 and HCCLM3 cells and correspondingly decreased when CCDC137 was knocked down in PLC/PRF/5 and JHH-7 cells (Fig. 4a, b; Supplementary Figure S3a, b), suggesting that CCDC137 positively regulates the AKT signaling pathway in HCC cells in vitro. Additionally, we examined AKT phosphorylation levels in murine HCC model specimens. Consistent with the results from in vitro HCC cell lines, the overexpression of CCDC137 promoted the phosphorylation of AKT at Ser473 and Thr308 (Fig. 4c; Supplementary Figure S3c). In contrast, CCDC137 depletion reduced AKT activation (Fig. 4d; Supplementary Figure S3c), which confirms that CCDC137 is a positive regulator of AKT signaling in vivo.

To further understand the importance of AKT signaling to the biological function of CCDC137 in HCC proliferation, we performed rescue assays using the AKT inhibitor MK-2206 to diminish the increased AKT phosphorylation level in CCDC137-overexpressing Huh7 cells (Fig. 4e). As expected, when the phosphorylation of AKT was inhibited, the proliferation-promoting ability of CCDC137 was significantly diminished (Fig. 4f, g), suggesting that the AKT signaling pathway is the major downstream effector of CCDC137. Furthermore, similar results were observed in the other CCDC137-overexpressing HCCLM3 cells, verifying that AKT signaling is the major downstream target of CCDC137 in HCC (Fig. 4h-j). Taken together, these results demonstrate that CCDC137 promotes HCC proliferation through the activation of the AKT signaling pathway.

### CCDC137 increases the cytoplasmic localization of *FOXM1*, *JTV1*, *LASP1* and *FLOT2* mRNAs to enhance their protein expressions

**Fig. 5:**
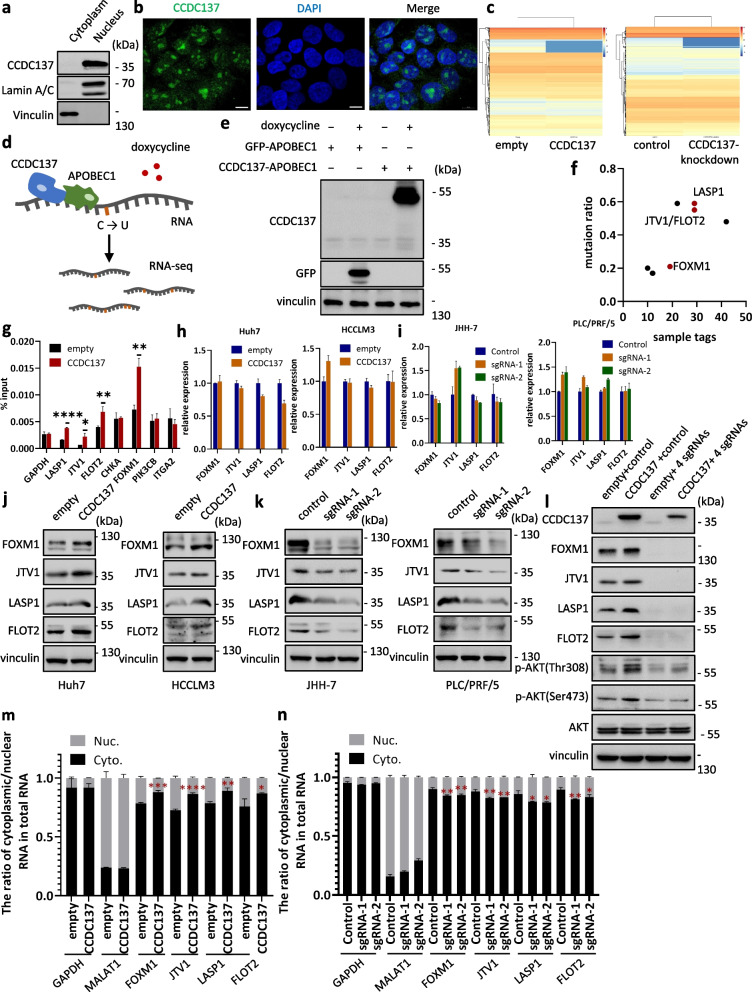
CCDC137 increases the cytoplasmic localization of *FOXM1*, *JTV1*, *LASP1* and *FLOT2* mRNAs to enhance their protein expressions. **a** Protein expression of CCDC137 in the cytoplasmic and nuclear fraction of JHH-7 cells. Lamin A/C as nuclear maker and vinculin as cytoplasmic marker. **b** Immunofluorescence microscopy shows that CCDC137 was mainly localized in the nucleus of JHH-7 cells. Scale bars: 10 μm. **c** Heat plots show the gene expressions in CCDC137-overexpressing Huh7 cells and CCDC137-knockdown PLC/PRF/5 cells. **d** Schematic illustration of STAMP. **e** GFP and CCDC137 protein expression in HCCLM3 cells transfected with GFP/CCDC137-APOBEC1 with or without doxycycline treatment. **f** Scatter diagram of AKT-related genes identified by STAMP. **g** RIP analysis of the interaction between CCDC137 and AKT-related genes identified by STAMP. CCDC137-overexpressing and control Huh7 cells were lysed and immunoprecipitated with anti-V5 affinity gel. The cell lysates and immunoprecipitants were detected by RT-qPCR. **h**, **i** Expressions of *FOXM1*, *JTV1*, *LASP1* and *FLOT2* mRNAs in CCDC137-overexpressing (**h**) and CCDC137-knockdown (**i**) cells. **j**, **k** Protein expressions of FOXM1, JTV1, LASP1 and FLOT2 in CCDC137-overexpressing (**j**) and CCDC137-knockdown (**k**) cells. **l** AKT phosphorylation levels at Ser473 and Thr308 in FOXM1, JTV1, LASP1 and FLOT2 simultaneously knockdown Huh7 cells.4 sgRNAs, sgRNAs target FOXM1, JTV1, LASP1 and FLOT2 were simultaneously transfected. (m, n) mRNA expressions of *FOXM1*, *JTV1*, *LASP1* and *FLOT2* in the cytoplasmic and nuclear fractionation of CCDC137-overexpressing Huh7 cells (**m**) and CCDC137 knockdown PLC/PRF/5 cells (**n**). GAPDH as cytoplasmic marker and MALAT1 as nuclear marker. Data were presented as mean ± s.d. of *n* = 3 independent experiments. *p* value: ^∗^*p* < 0.05, ^∗∗^*p* < 0.01, ^∗∗∗^*p* < 0.001, ^∗∗∗∗^*p* < 0.0001 by Student's *t*-test

Next, by detecting the nuclear and cytoplasmic fractions of proteins, we found that CCDC137 was mainly localized in the nucleus (Fig. 5a), which was further verified by immunofluorescence (IF) (Fig. 5b; Supplementary Figure S4). To further elucidate the mechanism of CCDC137-regulated AKT activation, we next sought to identify the downstream molecules of CCDC137. To determine whether CCDC137 affects the mRNA levels of genes regulating AKT activity, we performed transcriptome sequencing in CCDC137-overexpressing and CCDC137-knockdown cells. By analyzing sequencing data, we unexpectedly found that the overlap of genes with significantly changed expression between CCDC137-overexpressing and CCDC137-knockdown cells was very low and had no correlation with the AKT signaling pathway (Fig. 5c; Supplementary Figure S5), suggesting that nucleus-localized CCDC137 had a minor effect on mRNA expression and was unlikely to activate the AKT signaling pathway through regulation of mRNA expression.

To further explore the underlying mechanism of CCDC137, we attempted to identify CCDC137-interacting RNAs. We conducted STAMP (surveying targets by APOBEC-mediated profiling) by constructing the GFP-APOBEC1 and CCDC137-APOBEC1 fusion proteins [17, 18]. Cells overexpressing CCDC137-APOBEC1 or GFP-APOBEC1 were subjected to high through-put RNA-sequencing, and transcripts with mutations were resolved (Fig. 5d, e; Supplementary Figure S6a). Among these transcripts, we noted several AKT-related genes, four of which (FOXM1, JTV1, LASP1 and FLOT2 [25–28]) were verified by RNA immunoprecipitation (RIP) (Fig. 5f, g). Consistent with the transcriptome sequencing results, the mRNA levels of *FOXM1*, *JTV1*, *LASP1* and *FLOT2* were not significantly influenced by the overexpression or knockdown of CCDC137; however, the protein levels of FOXM1, JTV1, LASP1 and FLOT2 were upregulated in accordance with CCDC137 overexpression but downregulated when CCDC137 was knocked down (Fig. 5h-k; Supplementary Fig. 6b, c).

To verify the influence of FOXM1, JTV1, LASP1 or FLOT2 on the AKT signaling pathway, we knocked down their expressions in Huh7 cells. As shown in Supplementary Figure S6d and S6e, silencing FOXM1, JTV1, LASP1 or FLOT2 impaired the activation of the AKT signaling pathway as the phosphorylation levels of AKT at both Ser473 and Thr308 were decreased. To further define the role of FOXM1, JTV1, LASP1 and FLOT2 in CCDC137-induced AKT activation, we next knocked down FOXM1, JTV1, LASP1 and FLOT2 expression in CCDC137-overexpressing Huh7 cells. Surprisingly, the decreased expression of FOXM1, JTV1, LASP1 or FLOT2 could not completely diminish the activation of the AKT signaling pathway (Supplementary Figure S6f, g), but simultaneous depletion of FOXM1, JTV1, LASP1 and FLOT2 counteracted CCDC137-induced phosphorylation at both the Ser473 and Thr308 sites (Fig. 5l; Supplementary Figure S6h), suggesting that FOXM1, JTV1, LASP1 and FLOT2 work together in CCDC137-related AKT signaling pathway activation rather than one molecule playing a predominant role.

Next, we sought to explain the molecular mechanism underlying the regulation of FOXM1, JTV1, LASP1 and FLOT2 by CCDC137. As previously revealed, CCDC137 is a nucleus-localized oncogenic RBP and can regulate the protein expression levels of interacting molecules without affecting their mRNA levels, so we hypothesized that CCDC137 may influence the nuclear transport of *FOXM1*, *JTV1*, *LASP1* and *FLOT2* mRNAs. Using RT-qPCR analysis after nuclear/cytoplasmic fractionation, we found that cytoplasmic *FOXM1*, *JTV1*, *LASP1* and *FLOT2* mRNA expressions in CCDC137-overexpressing Huh7 cells were markedly increased compared with those in Huh7 cells transfected with empty vector, which was consistent with their elevated protein levels after CCDC137 overexpression (Fig. 5m). Correspondingly, in CCDC137 knockdown PLC/PRF/5 cells, cytoplasmic *FOXM1*, *JTV1*, *LASP1* and *FLOT2* mRNA expressions were decreased compared with those in the control cells, which was consistent with the reduced protein levels after CCDC137 was knocked down (Fig. 5n). These results demonstrate that CCDC137 positively regulates the cytoplasmic localization of *FOXM1*, *JTV1*, *LASP1* and *FLOT2* mRNAs.

Collectively, these data indicate that CCDC137 directly binds with *FOXM1*, *JTV1*, *LASP1* and *FLOT2* mRNAs and promotes their cytoplasmic localization to increase their protein expression without influencing mRNA levels. The upregulation of FOXM1, JTV1, LASP1 and FLOT2 proteins contributes synergistically to the activation of AKT signaling in HCC.

### CCDC137 promotes the cytoplasmic localization of downstream mRNAs through a noncanonical role of DGCR8

**Fig. 6:**
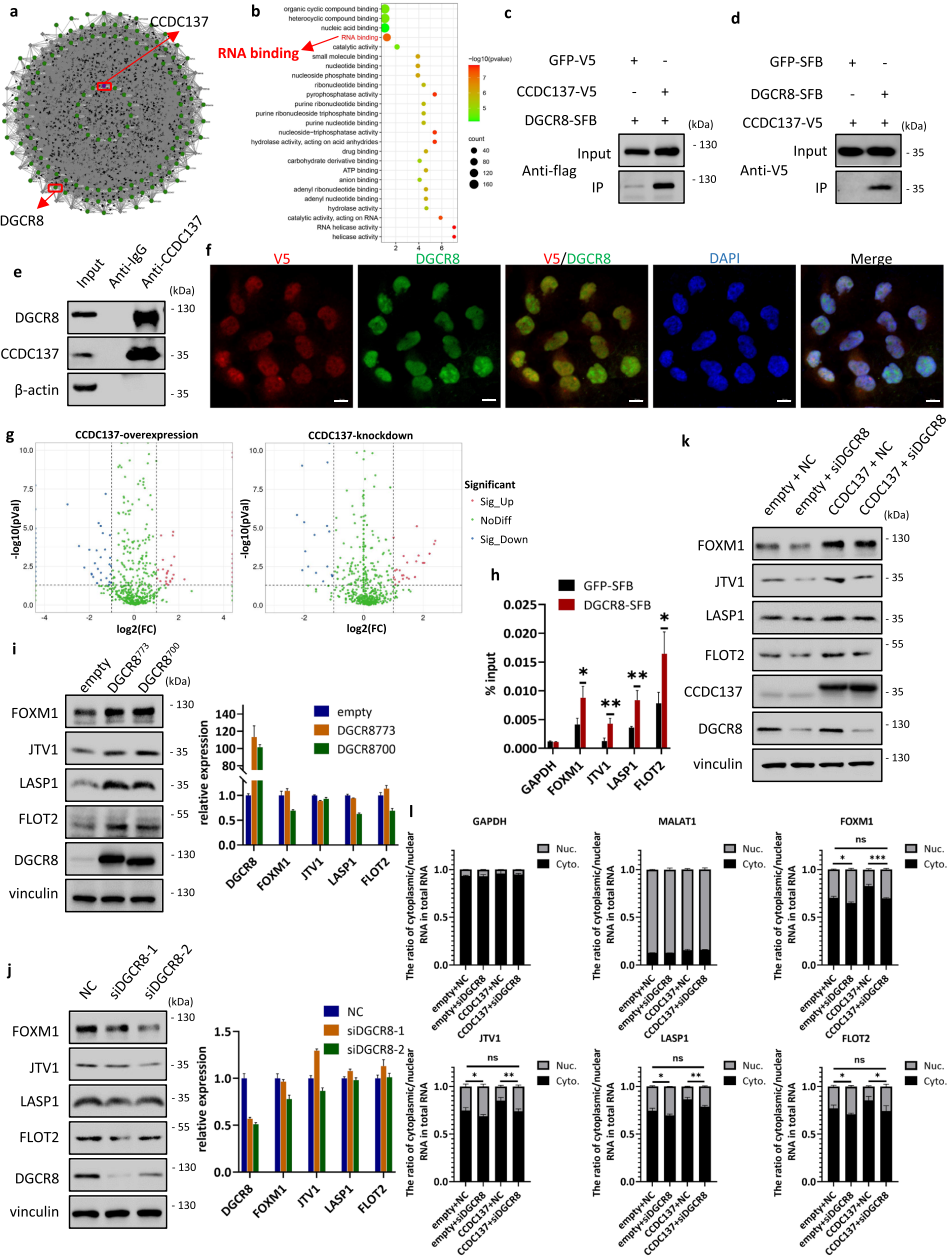
CCDC137 promotes the cytoplasmic localization of downstream mRNAs through a noncanonical role of DGCR8. **a**, **b** BIOPLEX network and gene ontology (GO) of the interacting proteins of CCDC137. **c**, **d** Co-IP of exogenous DGCR8 and CCDC137 in HEK293T cells. HEK293T cells were transfected with V5-tagged GFP or CCDC137 and SFB-tagged DGCR8 (**c**), SFB-tagged GFP or DGCR8 and V5-tagged CCDC137 (**d**). After 48 h, cell lysates were immunoprecipitated with V5 affinity gel (**c**) and S-protein Agarose (**d**). The cell lysates and immunoprecipitants were detected by western blot analysis with anti-Flag (**c**) and anti-V5 (**d**) antibodies. **e** Co-IP of endogenous DGCR8 and CCDC137 in JHH-7 cells. β-actin was used as negative control. **f** Immunofluorescence microscopy shows colocalization of V5 (red) and DGCR8 (green) in the nuclei (blue) of CCDC137-V5 and DGCR8-overexpressing Huh-7 cells. Scale bars: 10 μm. **g** Volcano plots show miRNA expressions fold change and significance in CCDC137-overexpressing Huh7 cells and CCDC137-knockdown PLC/PRF/5 cells. **h** RIP of exogenous DGCR8 interacted with FOXM1, JTV1, LASP1 and FLOT2 mRNAs in HEK293T cells. HEK293T cells were transfected with SFB-tagged GFP or DGCR8. After 48 h, cell lysates were immunoprecipitated with S-protein Agarose. RNA of cell lysates and immunoprecipitants was purified and detected by RT-qPCR. **i** Protein and mRNA expressions of FOXM1, JTV1, LASP1 and FLOT2 in DGCR8^773^ and DGCR8^700^-overexpressing Huh7 cells. **j** Protein and mRNA expressions of FOXM1, JTV1, LASP1 and FLOT2 in DGCR8 silenced Huh7 cells. **k** FOXM1, JTV1, LASP1 and FLOT2 protein expression in DGCR8 silenced Huh7 cells after CCDC137 overexpression. **l** Cytoplasmic and nuclear mRNA expression of *FOXM1*, *JTV1*, *LASP1* and *FLOT2* in DGCR8 silenced Huh7 cells after CCDC137 overexpression. GAPDH as cytoplasmic marker and MALAT1 as nuclear marker. NC, negative control. Data were presented as mean ± s.d. of *n* = 3 independent experiments. *p* value: ^∗^*p* < 0.05, ^∗∗^*p* < 0.01, ^∗∗∗^*p* < 0.001 by Student's *t*-test

Through bioinformatic analysis, we found that CCDC137 could bind with numerous proteins (Fig. 6a); Gene Ontology (GO) enrichment analysis revealed that RNA binding is most closely related to CCDC137 (Fig. 6b). Surprisingly, in these interacting proteins, we noticed that CCDC137 could bind with DGCR8, which was confirmed by both coimmunoprecipitation (Co-IP) and IF (Fig. 6c-f; Supplementary Figure S7). DGCR8 is an RNA-binding protein known to be part of the microprocessor that assists the RNase III enzyme Drosha in processing microRNAs (miRNAs) [29]. To determine whether CCDC137 could affect microRNA expression through its interaction with DGCR8, we performed small RNA-seq in CCDC137-overexpressing and CCDC137-knockdown cells. However, the results showed that the interaction of CCDC137 with DGCR8 had a minor effect on microRNA biogenesis (Fig. 6g; Supplementary Figure S8a-e).

A previous study has already reported the noncanonical role of DGCR8 in controlling the abundance of several mRNAs and lncRNAs [30]. Based on the role of CCDC137 in the cellular localization of *FOXM1*, *JTV1*, *LASP1* and *FLOT2* mRNAs, we then investigated whether DGCR8 participates in CCDC137 functionality. We first verified that DGCR8 could bind with *FOXM1*, *JTV1*, *LASP1* and *FLOT2* mRNAs using exogenous RIP (Fig. 6h). Next, we examined whether DGCR8 could regulate FOXM1, JTV1, LASP1 and FLOT2 expressions. Plasmids overexpressing full length DGCR8^773^ and truncated DGCR8^700^, which was unable to bind with Drosha and process microRNAs, were constructed and transfected into Huh7 cells. As shown in Fig. 6i and Supplementary Figure S8f, the protein expression levels of FOXM1, JTV1, LASP1 and FLOT2 were markedly increased, and their mRNA levels were unaffected in both DGCR8^773^ and DGCR8^700^ -overexpressing cells. Correspondingly, the protein expression levels, but not the mRNA levels, of FOXM1, JTV1, LASP1 and FLOT2 were significantly decreased when DGCR8 was knocked down by siRNAs (Fig. 6j; Supplementary Figure S8g). These data suggest that DGCR8 can positively regulate the protein expressions of FOXM1, JTV1, LASP1 and FLOT2 without affecting their mRNAs, which is independent of miRNA processing and consistent with the function of CCDC137.

To further elucidate the role of DGCR8 in CCDC137 functionality, we silenced DGCR8 in CCDC137-overexpressing Huh7 cells and examined the protein expression level and cytoplasmic expression of *FOXM1*, *JTV1*, *LASP1* and *FLOT2* mRNAs. In the context of CCDC137 overexpression, silencing DGCR8 eliminated the increased protein expression levels of JTV1, LASP1 and FLOT2 but only slightly reduced the elevated FOXM1 protein expression (Fig. 6k; Supplementary Figure S8h). By detecting the cytoplasmic/nuclear distribution, we found that DGCR8 depletion neutralized the increased cytoplasmic distribution of *FOXM1*, *JTV1*, *LASP1* and *FLOT2* mRNAs induced by CCDC137 overexpression (Fig. 6l), suggesting that DGCR8 mediates the positive regulation of the cytoplasmic localization of *FOXM1*, *JTV1*, *LASP1* and *FLOT2* mRNAs by CCDC137. Collectively, these results demonstrated a novel noncanonical role of DGCR8 in the cellular localization of mRNAs and indicated that CCDC137 promotes the cytoplasmic localization of downstream mRNAs through DGCR8.

## Discussion

**Fig. 7:**
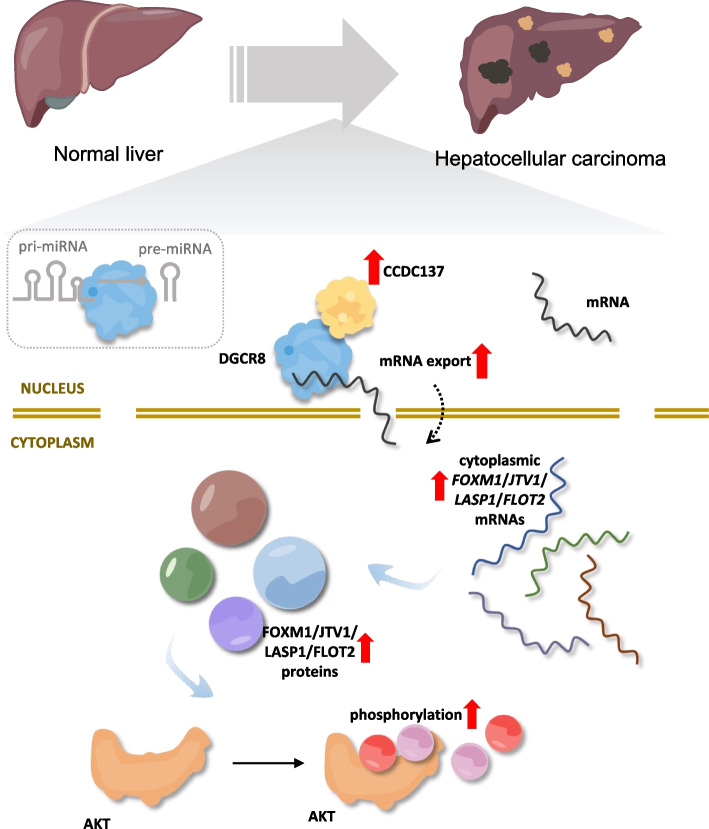
Schematic illustration of CCDC137 activates AKT signaling and promotes HCC proliferation through a noncanonical role of DGCR8 in the subcellular localization of targeting mRNAs

In this study, we identified a novel oncogenic RBP, CCDC137, whose expression was elevated in HCC tumor tissues compared with that in adjacent normal hepatic tissues. High CCDC137 expression was a robust predictive factor of poor prognosis of HCC patients. Functional studies demonstrated that CCDC137 promotes HCC proliferation through AKT signaling pathway activation and could be a potential therapeutic target of HCC. CCDC137 is localized to the nucleus, and we identified four interacting mRNAs, *FOXM1*, *JTV1*, *LASP1* and *FLOT2*, whose protein expression levels were positively regulated by CCDC137 without affecting their mRNA levels. The upregulation of FOXM1, JTV1, LASP1 and FLOT2 protein expressions mediated the activation of the AKT signaling pathway induced by CCDC137. CCDC137 could facilitate the cytoplasmic localization of *FOXM1*, *JTV1*, *LASP1* and *FLOT2* mRNAs, which resulted in upregulation of their protein expressions. We further found that CCDC137 could bind with another RNA binding protein, DGCR8, which could mediate the CCDC137-induced upregulation of FOXM1, JTV1, LASP1 and FLOT2 protein levels without affecting their mRNA levels and was independent of miRNA processing. Our study revealed a noncanonical role of DGCR8 in regulating mRNA cellular localization and establish a critical role of the CCDC137/DGCR8/AKT signaling axis in HCC tumorigenesis (Fig. 7).

The CCDC protein family is characterized by a shared coiled-coil domain, which is a highly-conserved super-helical protein motif consisting of wrapped alpha-helical peptides [31]. These proteins exhibit a wide range of expression in different tissues and play important functional roles in diverse physiological processes, such as neurological development and immune responses, and the most common role is in reproductive function [32]. For example, CCDC87 is important for sperm function [33] and CCDC38 regulates sperm flagellum biogenesis [34]. In addition, it’s increasingly evident that CCDC proteins are involved in the development and progression of cancers; for example, CCDC43 is an oncogenic factor in gastric cancer by promoting proliferation and metastasis [35], CCDC68 is a tumor suppressor of colorectal cancer through cell cycle arrest and growth inhibition [36], and CCDC106 suppresses lung adenocarcinoma by inducing c-Myc degradation [37]. To achieve such functionality, CCDC family proteins exhibit mechanistic diversity at the molecular level such as transcription, pre-mRNA splicing and translation [32]. Although expansive research has been carried out, much remains unknown about the biological functions and molecular mechanisms of CCDC family proteins. CCDC137 has been previously reported as a chromosome periphery protein and participates in HIV-1 infection [38]. Recent pan-cancer research revealed that CCDC137 is an oncogene and predicts poor prognosis in most cancer types [14]. In addition, CCDC137 is associated with immune infiltration and tumor progression in HCC [15]. Consistent with those results, this study identified CCDC137 as an oncogenic RBP in HCC and revealed the functional mechanism by which CCDC137 promotes mRNA cytoplasmic localization to activate AKT signaling.

DGCR8 is a microprocessor complex subunit that plays an essential role in microRNA biogenesis in eukaryotes. The primary functionality of DGCR8 in the microprocessor is recognizing and interacting with pri-miRNAs through the dsRNA-binding domains (dsRBDs) of DGCR8 [29]. However, further study has revealed that miRNAs are not the most abundant interacting substrate of DGCR8; substrate including mRNAs, small nucleolar RNAs (snoRNAs) and long noncoding RNAs (lncRNAs) [30] indicate that DGCR8 function is not limited to miRNA processing. Subsequently, a range of noncanonical roles of DGCR8 have been identified such as double-stranded RNA degradation [39], DNA damage response [40, 41], heterochromatin stabilization [42] and posttranscriptional regulation of mRNA like mRNA alternative splicing [43, 44]. These noncanonical functions of DGCR8 are independent of another microprocessor subunit, the RNase III enzyme Drosha, as well as canonical functions in miRNA processing. In our study, we found that DGCR8 could bind with CCDC137 and synergistically regulate FOXM1, JTV1, LASP1 and FLOT2 protein expressions independent of the interaction with Drosha, suggesting the existence of an alternative DGCR8 complex. Mechanistically, DGCR8 facilitates the cytoplasmic localization of *FOXM1*, *JTV1*, *LASP1* and *FLOT2* mRNAs without affecting their total mRNA levels. Based on these discoveries, we hypothesized that DGCR8 may play a role in mRNA nuclear export, but the specific mechanism needs to be further explored. Our research revealed a novel noncanonical function of DGCR8 and shedding light on further study of the microprocessor.

## Conclusions

In summary, this study established a correlation among an oncogenic RBP CCDC137, a novel non-canonical role of DGCR8 in mRNA subcellular localization and the AKT signaling, enriching the understanding of HCC-related RBPs and providing potential therapeutic targets.

## Supplementary Information

**Additional file 1.**

**Additional file 2: Supplementary Figure S1.** Expression level of CCDC137 in normal hepatocyte cell lines and HCC cell lines. (A) CCDC137 expression in normal hepatocyte cell lines (L-02 and THLE-2) and HCC cell lines (JHH-7, PLC/PRF/5, hepG2, SNU398, hep3B, SNU182, Huh7 and HCCLM3) was measured by Western Blot analysis. (B) Quantitative analysis of CCDC137/β-actin ratio. Data were presented as mean ± s.d. of *n* = 3 independent experiments.

**Additional file 3: Supplementary Figure S2.** The influence of CCDC137 expression on several HCC-related signaling pathways. Phosphorylation levels of JAK1 (a), STAT1 and STAT3 (b), GSK3β (c), ERK (d), S6K/S6, JNK and YAP (e) were measured by Western Blot in CCDC137-overexpressing and CCDC137-knockdown cells.

**Additional file 4: Supplementary Figure S3.** (a, b) Bar graphs of Fig. 4a (a), 4b (b). (c) Comparison of p-AKT (Thr308) and p-AKT (Ser473) positive area in Fig. 4c and 4d. Data were presented as mean ± s.d. *p* value: ^∗^*p* < 0.05, ^∗∗^*p* < 0.01, ^∗∗∗^*p* < 0.001 by Student's *t*-test.

**Additional file 5: Supplementary Figure S4.** Immunofluorescence microscopy of CCDC137 in Huh7, HCCLM3 and PLC/PRF/5 cells. Scale bars: 10 μm.

**Additional file 6: Supplementary Figure S5.** Analysis of transcriptome sequencing data in CCDC137-overexpressing and CCDC137-knockdown cells. The Venn diagram shows 13 (a) and 7 (c) genes with significant difference between CCDC137-overexpressing and CCDC137-knockdown cells. And the table listed the ID, name, fold change and expression levels of these genes (b, d).

**Additional file 7: Supplementary Figure S6.** The characterization of AKT-related and CCDC137-interacting genes. (a) Scatter plot shows the characterized genes with C to U mutations only in CCDC137-APOBEC1 expressing cells. (b, c) Bar graph of Fig. 5j (b) and 5 k (c). (d, e) AKT phosphorylation levels at Ser473 and Thr308 in FOXM1, JTV1, LASP1 and FLOT2-knockdown Huh7 cells and the bar graphs. (f, g) AKT phosphorylation levels at Ser473 and Thr308 in FOXM1, JTV1, LASP1 and FLOT2-knockdown Huh7 cells in the context of CCDC137 overexpression and the bar graphs. (h) Bar graph of Fig. 5l. Data were presented as mean ± s.d. *p* value: ^∗^*p* < 0.05, ^∗∗^*p* < 0.01, ^∗∗∗^*p* < 0.001 by Student's *t*-test.

**Additional file 8: Supplementary Figure S7.** Immunofluorescence microscopy of V5 and DGCR8 in CCDC137-V5 and DGCR8-overexpressing HCCLM3, JHH-7 and PLC/PRF/5 cells. Scale bars: 10 μm.

**Additional file 9: Supplementary Figure S8.** Analysis of small RNA-seq data in CCDC137-overexpressing and CCDC137-knockdown cells. The Venn diagram shows three (a) and one (c) microRNAs with significant difference between CCDC137 overexpression and knocked-down cells. And the table listed the name, sequence, fold change and expression levels of these genes (b, d). (e) Four miRNA expressions in CCDC137-overexpressing Huh7 cells and CCDC137-knockdown PLC/PRF/5 cells. (f–h) Bar graphs of Western Blot analysis in Fig. 6i (f), 6j (g) and 6 k (h). Data were presented as mean ± s.d. *p* value: ^∗^*p* < 0.05, ^∗∗^*p* < 0.01, ^∗∗∗^*p* < 0.001 by Student's *t*-test.

## Data Availability

The datasets supporting the conclusions of this article are available in the NCBI’s Gene Expression Omnibus (GEO) under accession code GSE218087.

## Abbreviations

RBPs: RNA binding proteins
HCC: Hepatocellular carcinoma
sgRNAs: Single guide RNAs
RT-qPCR: Real-time quantitative RT‒PCR
H&E: Hematoxylin and eosin
IHC: Immunohistochemistry
IF: Immunofluorescence
STAMP: Surveying targets by APOBEC-mediated profiling
RIP RNA: Immunoprecipitation
Co-IP: Coimmunoprecipitation
