# Correction: RNA‑binding protein CCDC137 activates AKT signaling and promotes hepatocellular carcinoma through a novel non‑canonical role of DGCR8 in mRNA localization

**DOI:** 10.1186/s13046-025-03612-3

**Published:** 2025-12-26

**Authors:** Shuang Tao, Shu-Juan Xie, Li-Ting Diao, Guo Lv, Ya-Rui Hou, Yan-Xia Hu, Wan-Yi Xu, Bin Du, Zhen-Dong Xiao

**Affiliations:** 1 Biotherapy Center, The Third Affiliated Hospital, Sun Yat-Sen University, Guangzhou, 510630 P.R. China; 2 Present address: Guangdong Provincial Key Laboratory of Malignant Tumor Epigenetics and Gene Regulation, Guangdong-Hong Kong Joint Laboratory for RNA Medicine, Medical Research Center, Sun Yat-Sen Memorial Hospital, Sun Yat-Sen University, Guangzhou, 510120 P.R. China; 3 Institute of Vaccine, The Third Affiliated Hospital, Sun Yat-Sen University, Guangzhou, 510630 P.R. China; 4 Guangdong Key Laboratory of Liver Disease Research, The Third Affiliated Hospital, Sun Yat-Sen University, Guangzhou, 510630 P.R. China; 5 Department of Pathology, Shanghai First Maternity and Infant Hospital, School of Medicine, Tongji University, Shanghai, 200092 P.R. China

**Correction: J Exp Clin Cancer Res 42, 194 (2023)**

**https://doi.org/10.1186/s13046-023–02749-3**

Following publication of the original article [1], the authors identified an error in Fig. 1e due to an unintentional oversight during figure assembly. The same loading control images were mistakenly used for both sample 11 and sample 12.

**Incorrect** Fig. 1

**Fig. 1:**
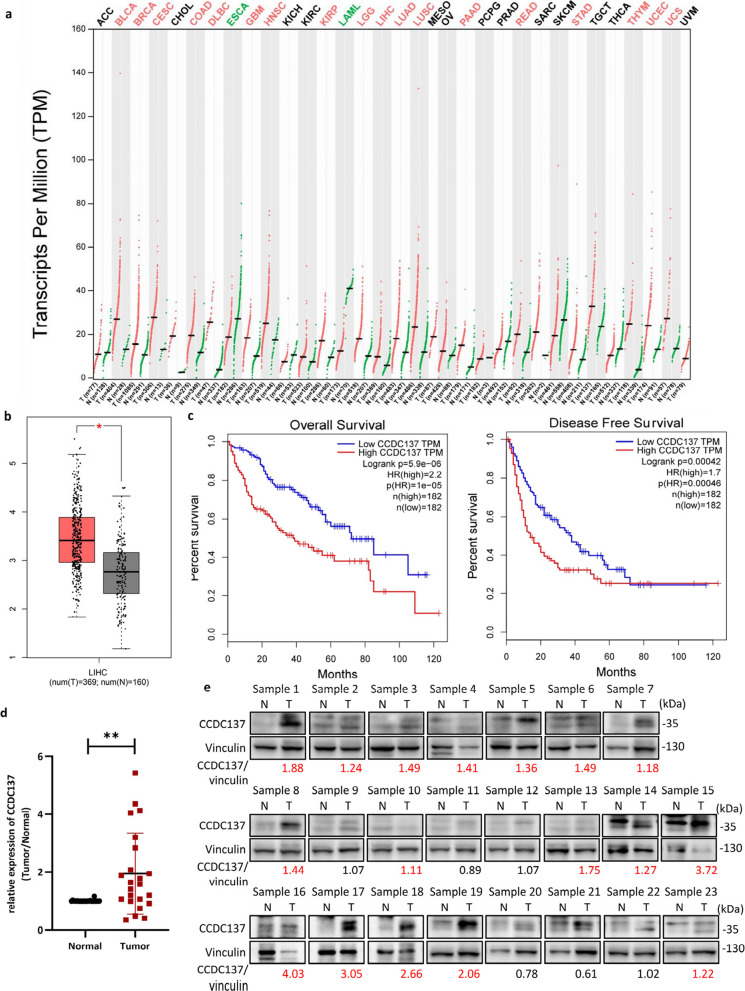
CCDC137 expression is elevated and associated with patient prognosis in HCC. **a, b, c** Analysis of CCDC137 expression from GEPIA (http://gepia.cancer-pku.cn/). **a** Dot plot shows CCDC137 expression in tumor samples (T, red) and paired normal tissues (N, green) across various tumor types. The X axis is the number of T and N for each tumor type. The Y axis is transcripts per million (TPM). Each dot represents expression level of samples. Labels in the top of the figure show different types of tumors marked with different color. Red, CCDC137 expression in T is significantly higher than N; green, CCDC137 expression in T significantly lower than N; black, have no significant difference. (log_2_FC cutoff = 0.5, *p* value cutoff = 0.01) **b** Box plot analysis of CCDC137 expression in 369 HCC tumor samples (T) and 160 normal tissues (N) (p* <0.05). (log_2_FC cutoff = 0.5, *p* value cutoff = 0.01) **c** Kaplan-Meier survival analysis of the correlation between CCDC137 expression and overall survival and disease-free survival in HCC. **d** *CCDC137* mRNA levels in 23 pairs of HCC tumor samples and corresponding adjacent non-tumor tissues. Data were presented as mean ± s.d. of n=3 independent experiments. *p* value: ^∗∗^*p* < 0.01 by paired Student's *t*-test. **e** CCDC137 protein levels in 23 pairs of HCC tumor samples and corresponding adjacent non-tumor tissues. The fold change of CCDC137/Vinculin ratio in tumor samples to normal samples over 1.1 were marked in red

**Correct **Fig. 1

**Fig. 1:**
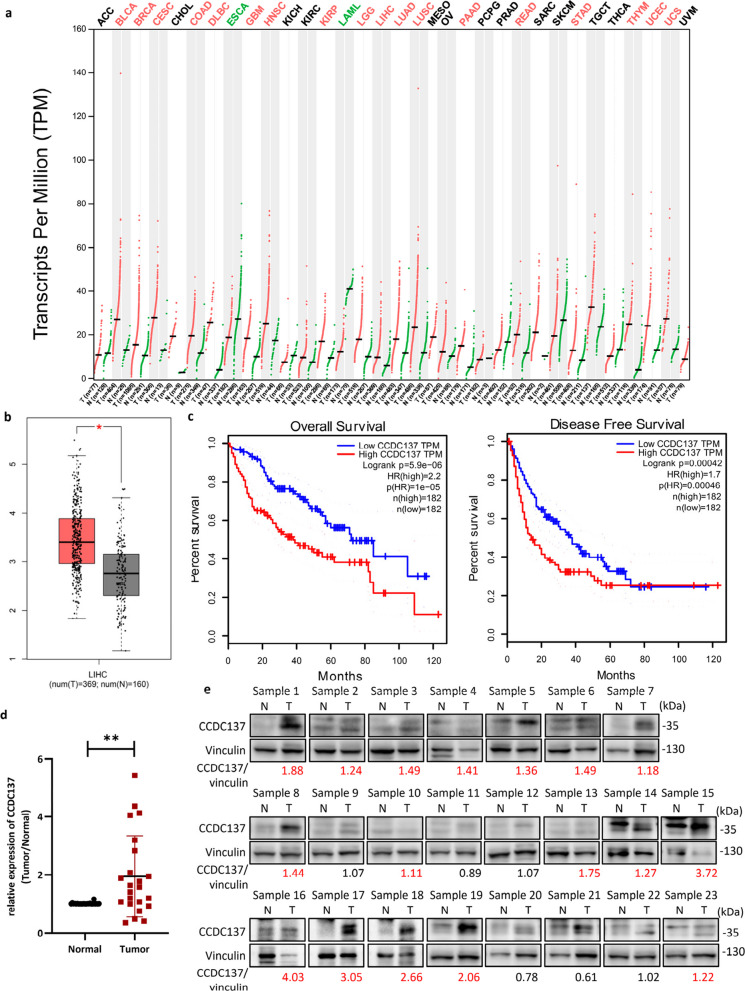
CCDC137 expression is elevated and associated with patient prognosis in HCC. **a, b, c** Analysis of CCDC137 expression from GEPIA (http://gepia.cancer-pku.cn/). **a** Dot plot shows CCDC137 expression in tumor samples (T, red) and paired normal tissues (N, green) across various tumor types. The X axis is the number of T and N for each tumor type. The Y axis is transcripts per million (TPM). Each dot represents expression level of samples. Labels in the top of the figure show different types of tumors marked with different color. Red, CCDC137 expression in T is significantly higher than N; green, CCDC137 expression in T significantly lower than N; black, have no significant difference. (log_2_FC cutoff = 0.5, *p* value cutoff = 0.01) **b** Box plot analysis of CCDC137 expression in 369 HCC tumor samples (T) and 160 normal tissues (N) (p* <0.05). (log_2_FC cutoff = 0.5, *p* value cutoff = 0.01) **c** Kaplan-Meier survival analysis of the correlation between CCDC137 expression and overall survival and disease-free survival in HCC. **d** *CCDC137* mRNA levels in 23 pairs of HCC tumor samples and corresponding adjacent non-tumor tissues. Data were presented as mean ± s.d. of n=3 independent experiments. *p* value: ^∗∗^*p* < 0.01 by paired Student's *t*-test. **e** CCDC137 protein levels in 23 pairs of HCC tumor samples and corresponding adjacent non-tumor tissues. The fold change of CCDC137/Vinculin ratio in tumor samples to normal samples over 1.1 were marked in red

The correction does not compromise the validity of the conclusions and the overall content of the article. The original article [1] has been corrected.
