# Supplementary figures and images for: RNA-binding protein CCDC137 activates AKT signaling and promotes hepatocellular carcinoma through a novel non-canonical role of DGCR8 in mRNA localization
Source: J Exp Clin Cancer Res. 2023 Aug 5;42:194. doi: 10.1186/s13046-023-02749-3 (PMC10403887; doi:10.1186/s13046-023-02749-3)

**a**

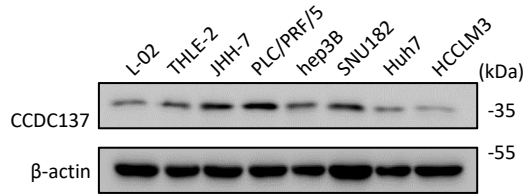

**b**

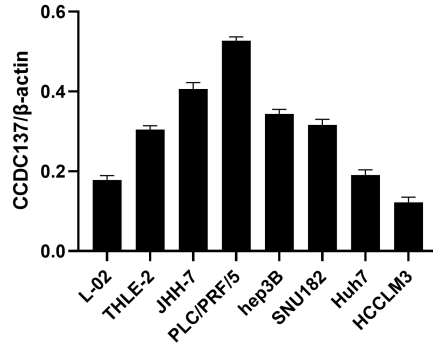

Supplement: Supplementary file 2 — Additional file 2: Supplementary Figure S1. Expression level of CCDC137 in normal hepatocyte cell lines and HCC cell lines. (A) CCDC137 expression in normal hepatocyte cell lines (L-02 and THLE-2) and HCC cell lines (JHH-7, PLC/PRF/5, hepG2, SNU398, hep3B, SNU182, Huh7 and HCCLM3) was measured by Western Blot analysis. (B) Quantitative analysis of CCDC137/β-actin ratio. Data were presented as mean ± s.d. of n = 3 independent experiments. [file 13046_2023_2749_MOESM2_ESM.pdf]

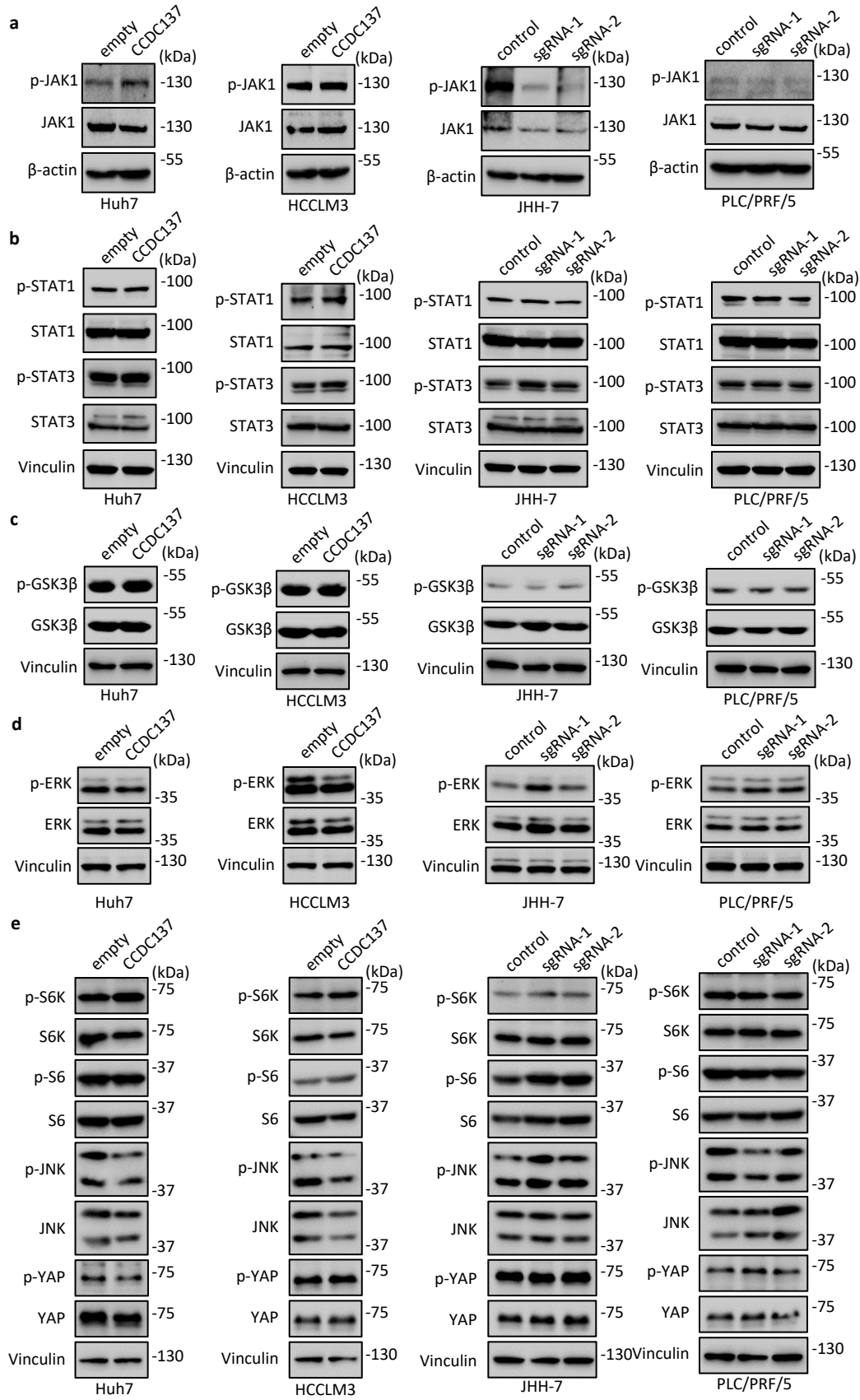

Supplement: Supplementary file 3 — Additional file 3: Supplementary Figure S2. The influence of CCDC137 expression on several HCC-related signaling pathways. Phosphorylation levels of JAK1 (a), STAT1 and STAT3 (b), GSK3β (c), ERK (d), S6K/S6, JNK and YAP (e) were measured by Western Blot in CCDC137-overexpressing and CCDC137-knockdown cells. [file 13046_2023_2749_MOESM3_ESM.pdf]

**a**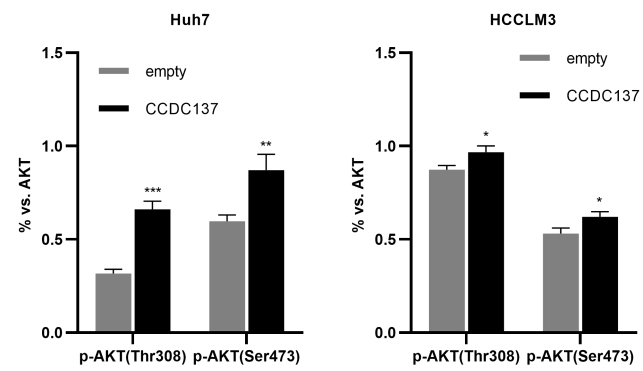**b**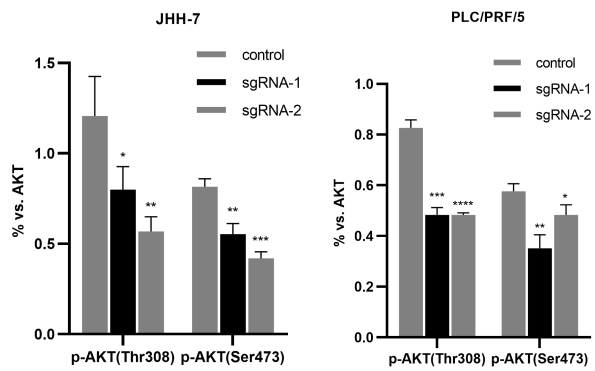**c**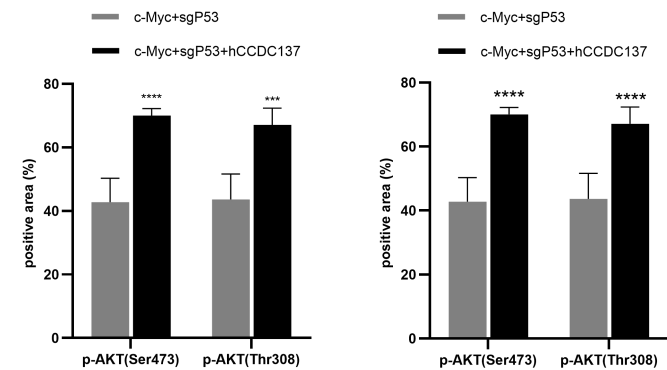

Supplement: Supplementary file 4 — Additional file 4: Supplementary Figure S3. (a, b) Bar graphs of Fig. 4a (a), 4b (b). (c) Comparison of p-AKT (Thr308) and p-AKT (Ser473) positive area in Fig. 4c and 4d. Data were presented as mean ± s.d. p value: ∗p < 0.05, ∗∗p < 0.01, ∗∗∗p < 0.001 by Student's t-test. [file 13046_2023_2749_MOESM4_ESM.pdf]

CCDC137

DAPI

Merge

Huh7

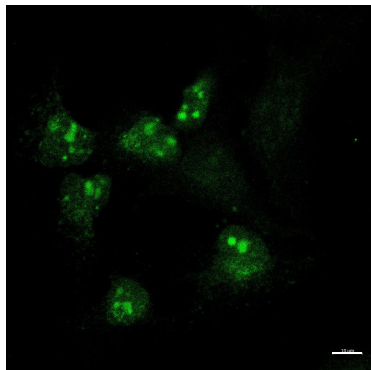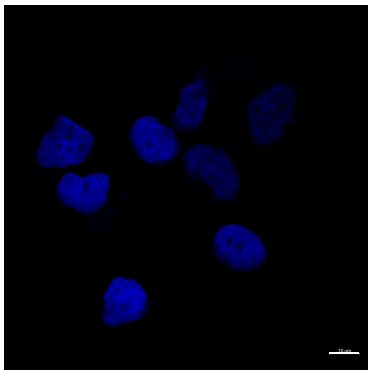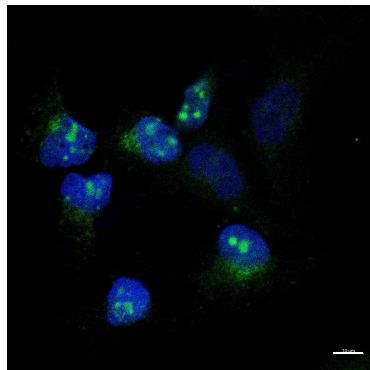

HCCLM3

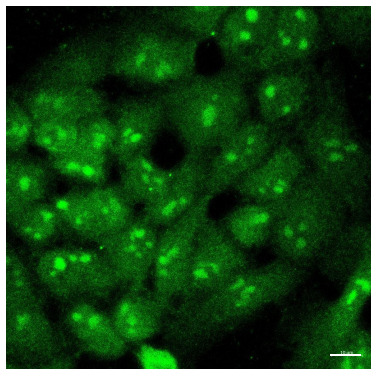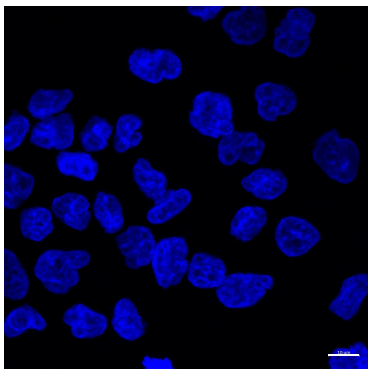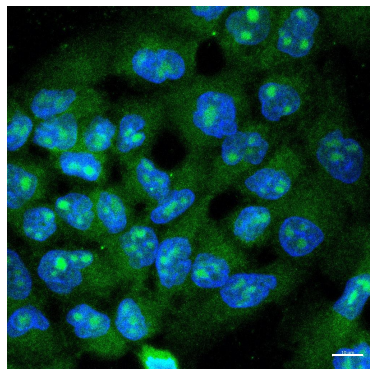

PLC/PRF/5

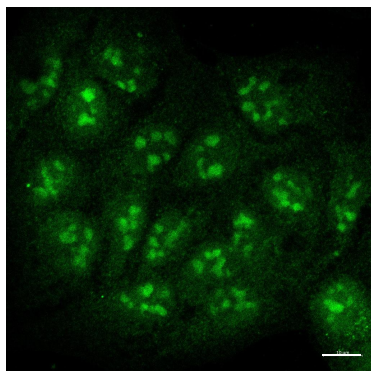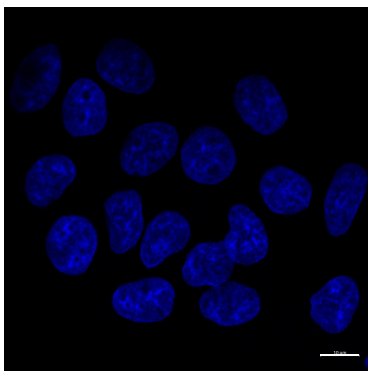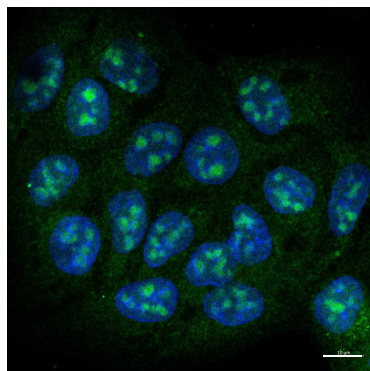

Supplement: Supplementary file 5 — Additional file 5: Supplementary Figure S4. Immunofluorescence microscopy of CCDC137 in Huh7, HCCLM3 and PLC/PRF/5 cells. Scale bars: 10 μm. [file 13046_2023_2749_MOESM5_ESM.pdf]

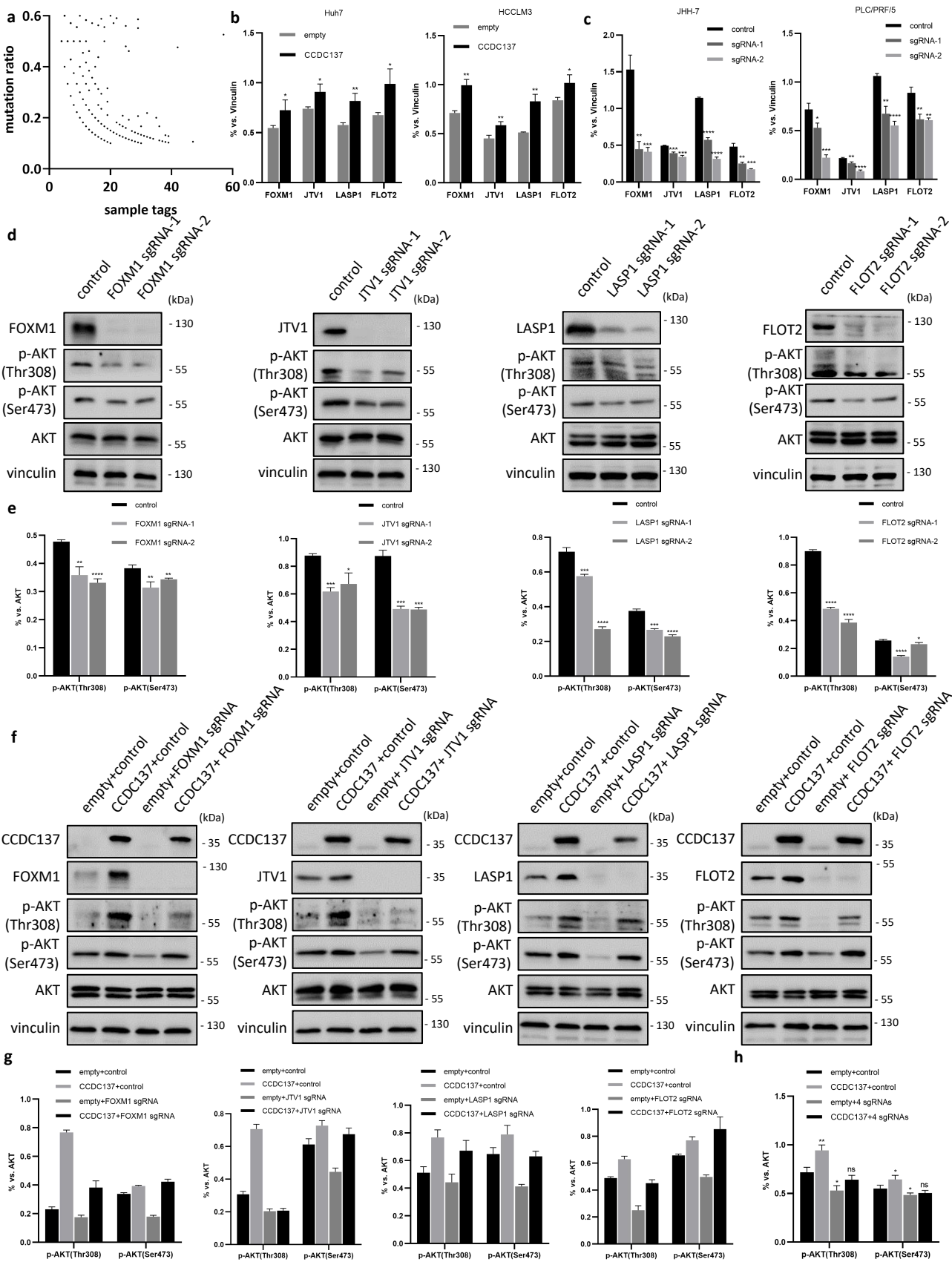

Supplement: Supplementary file 7 — Additional file 7: Supplementary Figure S6. The characterization of AKT-related and CCDC137-interacting genes. (a) Scatter plot shows the characterized genes with C to U mutations only in CCDC137-APOBEC1 expressing cells. (b, c) Bar graph of Fig. 5j (b) and 5 k (c). (d, e) AKT phosphorylation levels at Ser473 and Thr308 in FOXM1, JTV1, LASP1 and FLOT2-knockdown Huh7 cells and the bar graphs. (f, g) AKT phosphorylation levels at Ser473 and Thr308 in FOXM1, JTV1, LASP1 and FLOT2-knockdown Huh7 cells in the context of CCDC137 overexpression and the bar graphs. (h) Bar graph of Fig. 5l. Data were presented as mean ± s.d. p value: ∗p < 0.05, ∗∗p < 0.01, ∗∗∗p < 0.001 by Student's t-test. [file 13046_2023_2749_MOESM7_ESM.pdf]

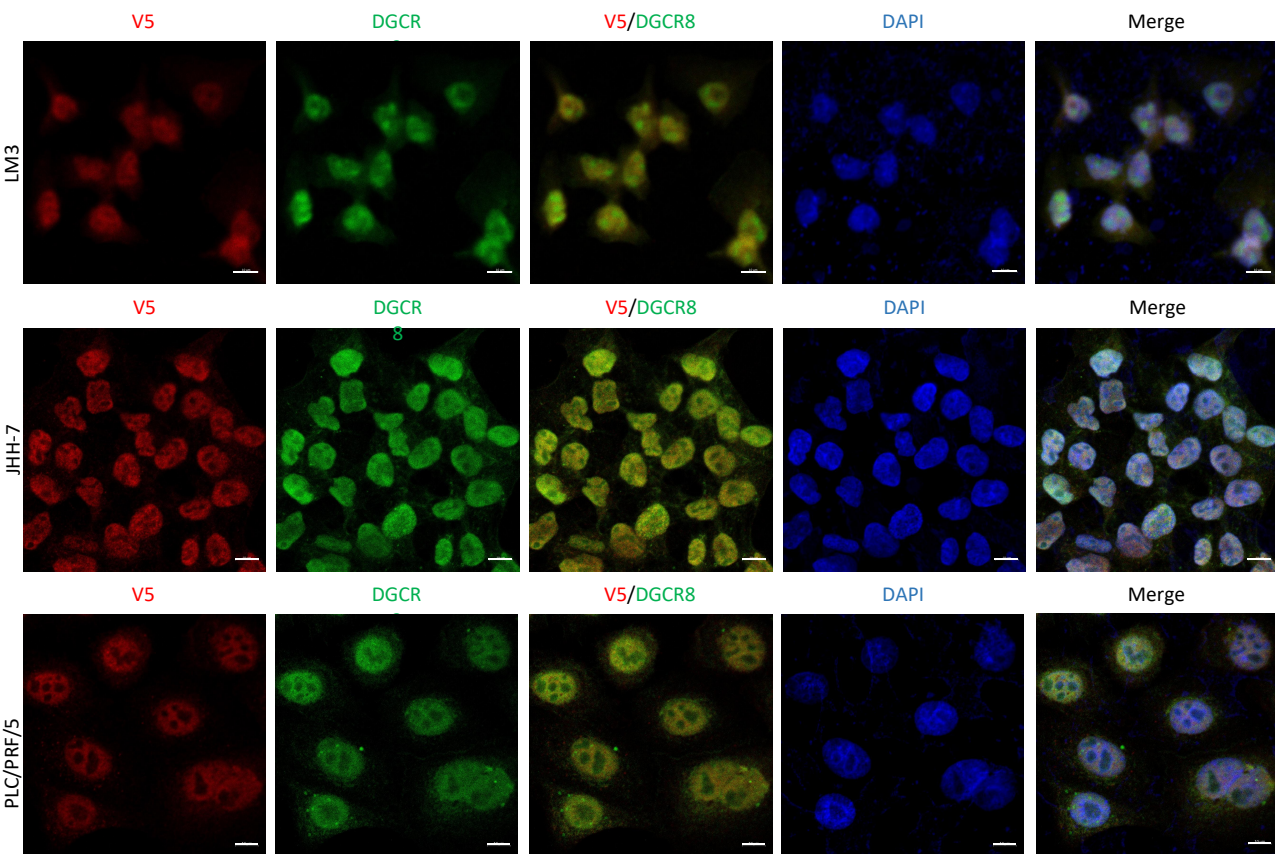

Supplement: Supplementary file 8 — Additional file 8: Supplementary Figure S7. Immunofluorescence microscopy of V5 and DGCR8 in CCDC137-V5 and DGCR8-overexpressing HCCLM3, JHH-7 and PLC/PRF/5 cells. Scale bars: 10 μm. [file 13046_2023_2749_MOESM8_ESM.pdf]

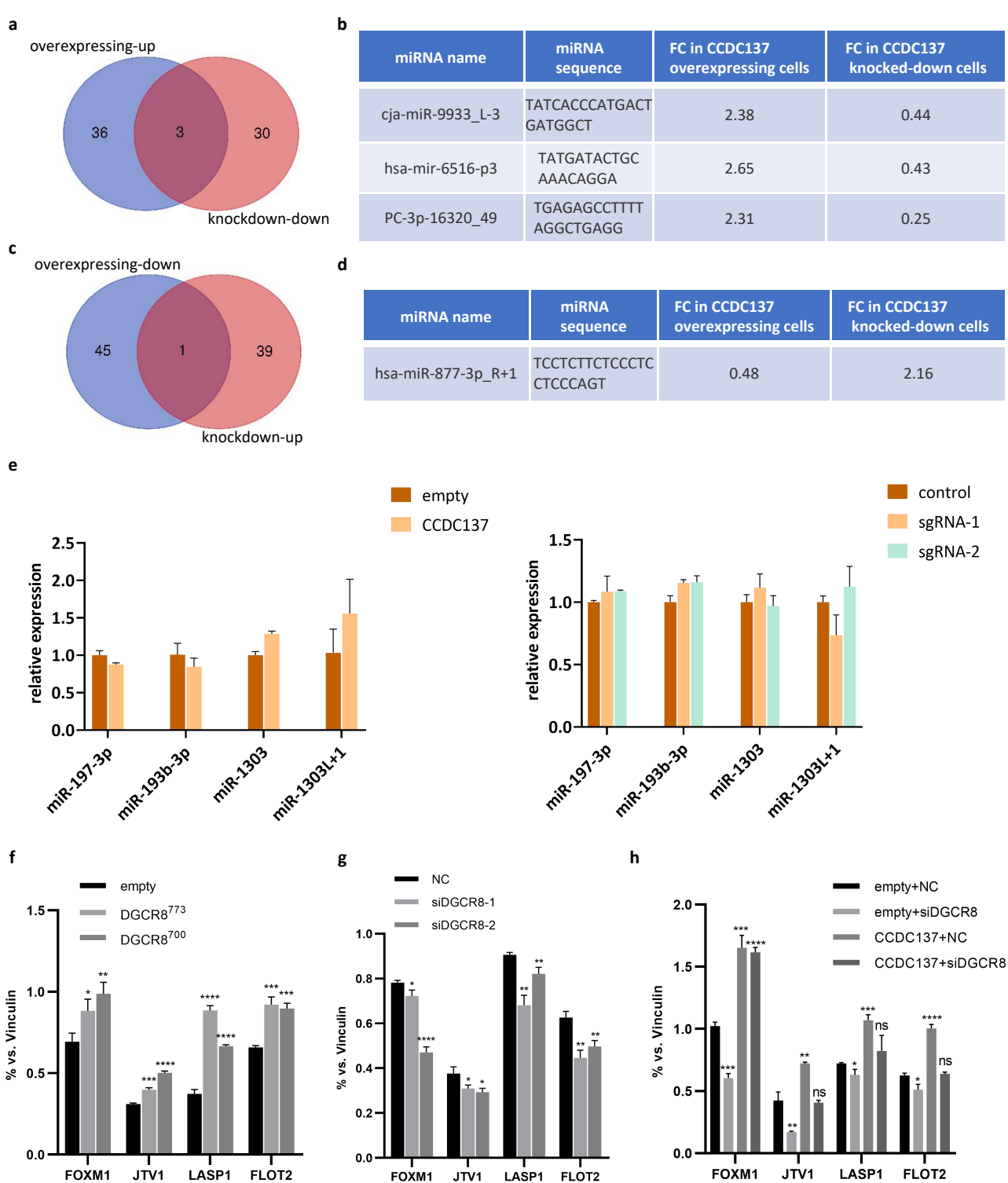

Supplement: Supplementary file 9 — Additional file 9: Supplementary Figure S8. Analysis of small RNA-seq data in CCDC137-overexpressing and CCDC137-knockdown cells. The Venn diagram shows three (a) and one (c) microRNAs with significant difference between CCDC137 overexpression and knocked-down cells. And the table listed the name, sequence, fold change and expression levels of these genes (b, d). (e) Four miRNA expressions in CCDC137-overexpressing Huh7 cells and CCDC137-knockdown PLC/PRF/5 cells. (f–h) Bar graphs of Western Blot analysis in Fig. 6i (f), 6j (g) and 6 k (h). Data were presented as mean ± s.d. p value: ∗p < 0.05, ∗∗p < 0.01, ∗∗∗p < 0.001 by Student's t-test. [file 13046_2023_2749_MOESM9_ESM.pdf]
