# Supplementary material for: RNA-binding protein CCDC137 activates AKT signaling and promotes hepatocellular carcinoma through a novel non-canonical role of DGCR8 in mRNA localization
Source: J Exp Clin Cancer Res. 2023 Aug 5;42:194. doi: 10.1186/s13046-023-02749-3 (PMC10403887; doi:10.1186/s13046-023-02749-3)
Supplement: Supplementary file 6 — Additional file 6: Supplementary Figure S5. Analysis of transcriptome sequencing data in CCDC137-overexpressing and CCDC137-knockdown cells. The Venn diagram shows 13 (a) and 7 (c) genes with significant difference between CCDC137-overexpressing and CCDC137-knockdown cells. And the table listed the ID, name, fold change and expression levels of these genes (b, d). [file 13046_2023_2749_MOESM6_ESM.pdf]

a

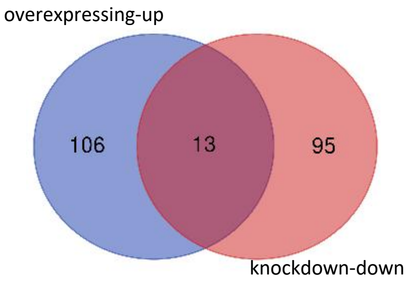

b

Gene ID	Gene name	FPKM in empty cells	FC in CCDC137-overexpressing cells	FPKM in control cells	FC in CCDC137-knockdown cells
ENSG00000178146	AL672207	0.94	2.73	2.40	0.35
ENSG00000242609	CCDC137	11.94	19.18	20.46	0.04
ENSG00000213625	LEPROT	2.43	6.23	14.66	0.19
ENSG00000134982	APC	0.74	2.65	2.15	0.28
ENSG00000198824	CHAMP1	0.41	14.50	10.02	0.12
ENSG00000258674	AC011448	0.48	26.08	10.89	0.21
ENSG00000259171	AL163636	0.65	3.73	4.29	0.38
ENSG00000123411	IKZF4	0.39	2.88	0.42	0.36
ENSG00000120784	ZFP30	0.20	3.93	2.67	0.26
ENSG00000167522	ANKRD11	3.88	3.65	10.70	0.34
ENSG00000254004	ZNF260	0.80	2.86	3.92	0.20
ENSG00000153165	RGPD3	0.16	3.37	0.50	0.27
ENSG00000139112	GABARAPL1	3.35	4.52	13.21	0.32

c

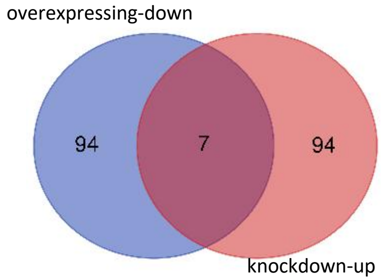

d

Gene ID	Gene name	FPKM in empty cells	FC in CCDC137-overexpressing cells	FPKM in control cells	FC in CCDC137-knockdown cells
ENSG00000124226	RNF114	23.40	0.39	4.64	2.65
ENSG00000178096	BOLA1	3.15	0.38	1.18	2.85
ENSG00000124767	GLO1	76.66	0.29	24.26	3.17
ENSG00000154608	CEP170P1	2.05	0.30	0.39	3.43
ENSG00000181027	FKRP	1.54	0.21	0.41	8.56
ENSG00000173402	DAG1	33.34	0.09	6.25	7.34
ENSG00000102780	DGKH	0.71	0.32	0.43	2.85
